# Modeling Interfacial Interaction between Gas Molecules and Semiconductor Metal Oxides: A New View Angle on Gas Sensing

**DOI:** 10.1002/advs.202203594

**Published:** 2022-09-18

**Authors:** Chenyi Yuan, Junhao Ma, Yidong Zou, Guisheng Li, Hualong Xu, Victor V. Sysoev, Xiaowei Cheng, Yonghui Deng

**Affiliations:** ^1^ Department of Chemistry, Department of Gastroenterology, Zhongshan Hospital of Fudan University State Key Laboratory of Molecular Engineering of Polymers Shanghai Key Laboratory of Molecular Catalysis and Innovative Materials, iCHEM Fudan University Shanghai 200433 China; ^2^ School of Materials and Chemistry University of Shanghai for Science & Technology Shanghai 200093 China; ^3^ Department of Physics Yuri Gagarin State Technical University of Saratov Saratov 410054 Russia

**Keywords:** adsorption, gas sensors, Knudsen diffusion, metal oxide semiconductors, surface catalysis

## Abstract

With the development of internet of things and artificial intelligence electronics, metal oxide semiconductor (MOS)‐based sensing materials have attracted increasing attention from both fundamental research and practical applications. MOS materials possess intrinsic physicochemical properties, tunable compositions, and electronic structure, and are particularly suitable for integration and miniaturization in developing chemiresistive gas sensors. During sensing processes, the dynamic gas–solid interface interactions play crucial roles in improving sensors’ performance, and most studies emphasize the gas–MOS chemical reactions. Herein, from a new view angle focusing more on physical gas–solid interactions during gas sensing, basic theory overview and latest progress for the dynamic process of gas molecules including adsorption, desorption, and diffusion, are systematically summarized and elucidated. The unique electronic sensing mechanisms are also discussed from various aspects including molecular interaction models, gas diffusion mechanism, and interfacial reaction behaviors, where structure–activity relationship and diffusion behavior are overviewed in detail. Especially, the surface adsorption–desorption dynamics are discussed and evaluated, and their potential effects on sensing performance are elucidated from the gas–solid interfacial regulation perspective. Finally, the prospect for further research directions in improving gas dynamic processes in MOS gas sensors is discussed, aiming to supplement the approaches for the development of high‐performance MOS gas sensors.

## Graphical abstract

Chemiresistive gas sensors are important sensing devices, whose operation matures from the interface interaction and heterogeneous catalysis between gas molecules and metal oxide semiconductors (MOS). Modeling the dynamic processes of gas molecules over porous sensitive MOS layers including gas adsorption, desorption, and diffusion, is a new and complementary view angle to understand gas sensing mechanism and develop high‐performance gas sensors.

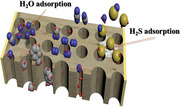

## 1 Introduction

Nowadays, with the rapid development of nanotechnology, novel sensors are playing more and more important roles in various fields including environmental monitoring,^[^ ^1^ ^]^ industrial manufacturing,^[^ ^2^ ^]^ military,^[^ ^3^ ^]^ and public safety and health.^[^ ^4^ ^]^ The principle of gas sensors is mainly based on the chemiresistance effect of sensing materials toward different gas molecules, which can convert the concentration variation of tested gases into the corresponding signals.^[^ ^5^ ^]^ Notably, metal oxide semiconductors (MOSs) have become a kind of the most widely used sensing materials for the detection of gas molecules due to their favorable physical/chemical properties as well as tunable compositions, unique micro‐/nanostructures, diversified morphologies, and suitability for device integration and miniaturization.

Ever since the application of MOS materials was pioneered in gas sensing,^[^ ^6^ ^]^ numerous studies have been carried out on the synthesis and sensing application of gas‐sensitive materials with different morphologies or compositions.^[^ ^7^ ^]^ However, to date, several important scientific issues such as the regulation principles of sensing performance have not been fully addressed, and this restricts the application and exploitation of high‐performance gas sensors. Although some sensitive materials optimization strategies have been reported, including surface modification (e.g., porous, and low‐dimensional materials),^[^ ^7b,c,h,i^ ^]^ heterojunctions (doping of other MOS materials),^[^ ^7a,f,g,j^ ^]^ loading of noble metals (spill‐over effect),^[^ ^7d–f^ ^]^ and light activation,^[^ ^5^, ^7^ ^]^, the significant impacts of other factors, such as framework microenvironment and interfacial interaction on the gas sensing performance, have not been attracted sufficient attention, and thus they should be considered and explained in depth.

In reality, in a typical sensing process of MOS gas sensors, the injected gas molecules can rapidly diffuse and fill the whole test chamber, and meanwhile, some of them are adsorbed onto the surface of sensitive materials, causing the resistance or conductivity variation of sensitive MOS, and this process is termed as “response”.^[^ ^8^ ^]^ In other words, the adsorption of tested gas molecules marks the “start” of the sensing process, which is highlighted as a critical step for the sensing detection of gases. On the other hand, desorption also occurs simultaneously as a reversible process, and some of the adsorbed gas molecules are released into the air again. The remaining adsorbed molecules then undergo a coupled diffusion–reaction (DR) process,^[^ ^9^ ^]^ where the molecules can diffuse into deeper areas of the sensing materials and react with exposed active sites to change the resistance. The consumption and/or transformation of the gas molecules can cause dramatic changes in resistance to boost the responses accordingly. After equilibrium for a period and then the evacuation of the tested gas, the initial situations of sensing materials are restored in air, during which the tested gas together with derivative molecules are desorbed from the MOS surface into the air. Therefore, adsorption, desorption, and diffusion of gases in the whole testing process should be precisely investigated to recognize the sensing mechanisms. However, a lot of mathematical and physical models are essential for mechanism explanation through analyzing the adsorption, desorption, and diffusion of gases,^[^ ^10^ ^]^ and this was usually neglected in most previous studies involving the design and synthesis of sensing materials. Although some studies about gas sensing have touched on these aspects, few reviews have systematically combined the intrinsic relationships of adsorption, desorption, and diffusion of gases with the gas responses of MOS sensors.

**Figure 1:**
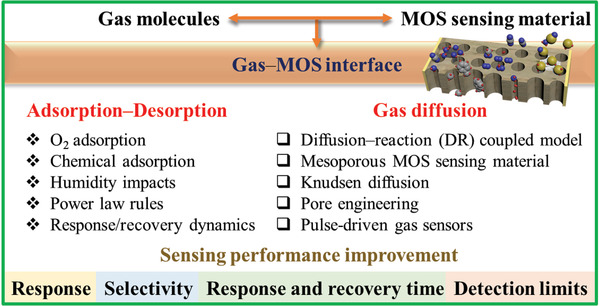
Overall scheme for the dynamic processes of adsorption/desorption and gas diffusion at the gas–MOS interface and gas sensing performance improvement for MOS gas sensors.

Herein, this review focuses on the close linkage between the dynamic motions of gas molecules and the sensing mechanisms, and the impacts of adsorption and desorption on gas sensing are explained in detail. According to different models, the adsorption mechanisms on MOSs are generally divided into three types based on oxygen adsorption, chemical adsorption, and physical adsorption. The power law and response/recovery processes have close correlations with the adsorption/desorption of gases, which are further discussed for gas sensing mechanisms from this point of view. In addition, some representative diffusion models for gas sensing are also introduced, including Gardner's models, Yamazoe's models, and other modified models. The related classical mathematical and physical theories are given out together with precise derivations and inferences to lay a solid foundation for a clear description of the relevant mechanisms. The related research directions are illustrated in **Figure** **1**, and it is expected that this review can boost a close combination of the conventional material‐based sensing research with the assistance of theory‐based mathematical or physical derivations, thus expanding the field of gas sensing to a broader horizon.

## 2 Adsorption and Desorption

### 2.1 Classical Adsorption and Desorption Theories

Generally, adsorption is defined as the adhesion of particles from a fluid to a surface. According to the definition of the International Union of Pure and Applied Chemistry (IUPAC), adsorption means the “increase in the concentration of a substance at the interface of a condensed and a liquid or gaseous layer owing to the operation of surface forces”, and desorption is the opposite process of adsorption. Adsorption is conventionally divided into physisorption and chemisorption, to be distinguished primarily by activation energy, ordinarily less than 25.116 kJ mol^−1^ and higher than 62.79 kJ mol^−1^, respectively. Through van der Waals and polarization interactions during the physisorption process, the electronic structure of the particle is barely perturbed upon adsorption. Differently, chemisorption happens through forming chemical bonds between adsorbate and adsorbent, involving charge carrier exchange.^[^ ^11^ ^]^

The adsorption of particles, that is, the amount of adsorbate on the surface of the adsorbent, can be precisely described with sorption isotherms based on various models at a constant temperature. The common isotherms are mainly interpreted with Henry's adsorption isotherm, Freundlich equation, Langmuir equation, and Brunauer–Emmett–Teller (BET) theory. Henry's adsorption isotherm, named after British chemist William Henry, is one of the simplest models. The model is based on Henry's law, in which the amount of dissolved gas in a solution is proportional to its partial gas‐phase pressure. Therefore, the number of gas molecules adsorbed onto the surface is also proportional to the partial gas pressure in Henry's adsorption isotherm. As shown in Equation (1), *X*, *K*_H_, and *P* stand for surface coverage, Henry's adsorption constant, and partial pressure, respectively. All the isotherms mentioned above follow the linear correlation at low pressures, and Henry's adsorption isotherm is only valid for the adsorption at low surface coverages.

(1) $$X=K_{H}P$$

Freundlich equation, named after German chemist Herbert Freundlich, is an expansion of Henry's adsorption isotherm. It is empirically estimated that unit adsorbing capacity is proportional to a power of equilibrium pressure. Freundlich adsorption isotherm can be expressed as Equation (2), in which *x*, *M*, and *p* stand for the mass of adsorbate, the mass of adsorbent, and equilibrium pressure of adsorbate, respectively, and *K* and *n* remain constants for a specific adsorbent at a certain temperature. At high temperatures, *n* is close to 1, and the equation thus returns to Equation (1). Since it is usually appropriate to fit isotherms on a theoretical basis via empirical estimation, certain limitations still exist in the derived equations. The most significant limitation of the Freundlich equation is that it is not applicable at high pressures. In this case, it is thought to achieve an adsorption saturation, which is quite different from practical experiments. For example, in the case of H_2_S adsorption on activated carbon, different deviations can be obtained at high and low pressures from the isotherm, respectively.^[^ ^12^ ^]^

(2) $$\frac{x}{M}=Kp^{\frac{1}{n}}$$

In 1918, American physicist and chemist Irving Langmuir elucidated a semi‐empirical adsorption isotherm based on kinetic assumptions and statistical thermodynamics, which is defined as Langmuir adsorption. Due to its simplicity and validity in various adsorption situations, up to now, it is considered one of the most common isotherm equations. The equation is mainly based on four hypotheses as follows:^[^ ^13^ ^]^

a) All adsorption sites are equivalent, each of which is only available for one gas molecule;
b) the surface of the adsorbent is homogeneous, and no interaction exists among adsorbate molecules;
c) no phase transitions happen during adsorption;
d) only one monolayer of gas molecules is formed during adsorption.

Based on kinetic derivation, thermodynamic or statistical mechanical derivation, the Langmuir equation is usually expressed as Equation (3), where *θ*, *K*, and *P* stand for surface coverage, the equilibrium constant for adsorption/desorption reaction, and partial pressure of the gas, respectively. At low pressures, *θ* ≈ *KP*, while at high pressures, *θ* ≈ 1. Burke et al. elucidated that the enthalpies of adsorption could be considered for model selection, and it was a common misconception to choose an adsorption model according to the best fitting data.^[^ ^14^ ^]^

(3) $$\theta =\frac{KP}{1+KP}$$

Although the Langmuir equation has a rather wide application range, it is still invalid in multilayer conditions, in which gas molecules are adsorbed on the last layer of already adsorbed molecules. In 1938, American chemists Stephen Brunauer, Paul Emmett, and physicist Edward Teller put forward a novel isotherm (BET theory), which takes multilayer adsorption into account based on the following four hypotheses:^[^ ^15^ ^]^

a) Physisorption of gas molecules happens on a solid with infinite layers;
b) gas molecules in adjacent layers merely have interactions, and the Langmuir theory can be applied to every single layer of gas molecules;
c) the adsorption enthalpy for the first layer is constant, which is much higher than that for the other layers;
d) the adsorption enthalpy for higher layers is equal to the enthalpy of liquefaction.

The BET equation can be eventually derived as Equation (4), in which *x* is the partial pressure of the adsorbate molecules, *v* is the volume of adsorbate at standard conditions for temperature and pressure (STP), and *v*_mon_ is the volume of adsorbate at STP required to form a monolayer, while *c* is the equilibrium constant for adsorption/desorption reaction.

(4) $$\frac{x}{v(1-x)}=\frac{1+x(c-1)}{v_{m\mathrm{on}}c}$$

Although the BET theory takes the multilayer adsorption into account, the adsorption experiments are usually carried out at the boiling point of N_2_ (77 K), which is inapplicable for conventional gas sensing experiments with an operating temperature of several hundred Kelvin degrees. Therefore, the adsorption of gas molecules at higher layers contributes little to gas‐sensing responses. As a result, the Langmuir model is one of the most frequently used adsorption models in practical gas sensing research.^[^ ^16^ ^]^ Moreover, the adsorption/desorption mechanism can be applied in surface reactions of gas molecules as well. Irving Langmuir (1921) and British chemist Cyril Hinshelwood (1926) elucidated a reaction mechanism (Langmuir–Hinshelwood model or L–H model), showing that two molecules were adsorbed on neighboring sites and then underwent a bimolecular reaction.^[^ ^17^ ^]^ The eventual equilibrium constant correlates with the surface coverage of the reactant molecules on the materials and the rate constant of the adsorption/desorption process. In 1938, British chemists Dan Eley and Eric Rideal elucidated another adsorption mechanism (Eley–Rideal model, or E–R model), in which only one of the molecules adsorbed onto the material surface reacts with the other unabsorbed one in the gas phase.^[^ ^18^ ^]^ The eventual equilibrium constant correlates with the surface coverage of A molecules adsorbed on the material. In 1954, Dutch chemists Mars and Van Krevelen further elucidated a novel adsorption‐based catalytic mechanism (MvK mechanism,^[^ ^19^ ^]^ or redox/regenerative mechanism),^[^ ^20^ ^]^ in which the catalyst lattice reacting with the reactant was depleted. Thus, a surface vacancy was created, which might be later supplemented by further reconfiguration. In the MvK mechanism, radicals are generally formed on the surface (e.g., radical coupling of methane) before those lattice ingredients are released to the gas phase and depleted.^[^ ^21^ ^]^

**Figure 2:**
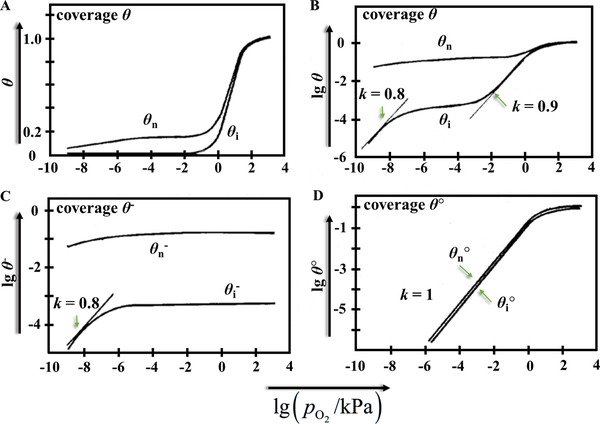
The Wolkenstein isotherms. The coverages of chemisorbed acceptor‐like species on n‐type ZnO films are presented for the highly‐compensated case (index i) and for the uncompensated case (index n), depending on the oxygen partial pressure. A) *Θ*–lg *p* presentation and B) lg *Θ*–lg *p* presentation of the total coverage, *Θ*(*p*). C,D) Logarithmic presentations of the coverages of the charged strong‐chemisorbed and neutral weak‐chemisorbed species. Reproduced with permission.^[^ ^25^ ^]^ Copyright 1993, Elsevier.

Furthermore, the Langmuir isotherm has been considered in the framework of electron adsorption theory by Wolkenstein who updated Equation (3) by introducing the dependence of *K* not only on the temperature but also on the Fermi level in the material.^[^ ^22^ ^]^ In other words, it means the adsorption is managed by the electrical properties of the solid state and its surface states, which primarily appeared in the material's forbidden gap. Wolkenstein's isotherm yields possibly the most full explanation for adsorbate–adsorbent interaction. As shown by Geistlinger, the electron theory allows one to observe Henry, Freundlich, and logarithmic isotherms as partial cases (**Figure** **2**).^[^ ^23^ ^]^ For example, numerical research could properly explain the experimentally observed gas response (*S*)‐to‐grain size (*D*) curves of *S*–1/*D* by applying this theory.^[^ ^23^ ^]^ Also, the consideration of adsorbed species as a surface donor‐ or acceptor‐like defects in metal oxides made it possible to calculate the variations of their resistivity upon exposure to gases of reducing and oxidizing nature, at least under conditions when the Fermi level is pinned at the corresponding states.^[^ ^24^ ^]^

**Figure 3:**
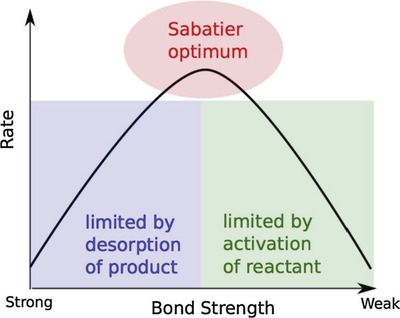
Schematic representation of the qualitative Sabatier–Balandin principle. Reproduced with permission.^[^ ^26^ ^]^ Copyright 2015, Elsevier.

In 1920, French chemist Paul Sabatier elucidated a principle, in which the interaction between catalyst and reactants in a catalytic reaction should be moderate (Sabatier optimum). Otherwise, it is hard to activate the reactants with weak interaction and desorb the product molecules with strong interaction.^[^ ^26^ ^]^ In 1969, Balandin further elucidated that the correlation between the reaction rate and bond strength of catalysts could form a volcano curve with a maximum peak (**Figure** **3**).^[^ ^27^ ^]^ The Sabatier–Balandin principle has been widely applied to qualitatively understand how to design the optimum catalyst.

### 2.2 Impacts of Adsorption and Desorption on MOS Gas Sensing

It's well known that the responses of MOS gas sensing materials are displayed as the changes of resistance when exposed to different gases. In practical gas sensing experiments, physical adsorption is relatively weak and susceptible to conditions (such as humidity and temperature), while chemical adsorption/desorption dominates the sensing process and is more stable based on strong bond interactions. In other words, chemical bonds are formed between adsorbed gas molecules and MOS materials in most cases, leading to a rapid change of resistance. However, due to the universal existence of oxygen in testing environments, the adsorption of oxygen molecules is a quite important and non‐negligible process, which is commonly separate from chemical adsorption.^[^ ^28^ ^]^ Moreover, combining the theory of depletion layer derived from the oxygen adsorption model with gas adsorption and reaction, the theory of power law for MOS gas sensors was elucidated, showing a power law correlation between resistance and partial pressure of gas molecules.^[^ ^10g^ ^]^ Therefore, in practical gas sensing testing, the chemical adsorption of oxygen and tested gas as well as the desorption of product molecules have a significant impact on the response and recovery of gas sensors, which can be further manipulated in boosting sensing properties and increasing selectivity of the sensing materials towards some specific gases.

#### 2.2.1 Oxygen Adsorption Models

**Figure 4:**
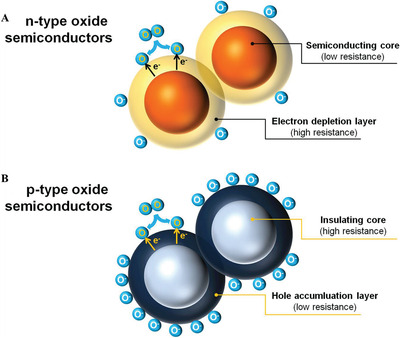
Formation of electronic core–shell structures in A) n‐type and B) p‐type oxide semiconductors. Reproduced with permission.^[^ ^34^ ^]^ Copyright 2014, Elsevier.

When the MOS material is exposed to dry air, nitrogen, and oxygen surely produce impacts on gas–solid interaction.^[^ ^29^ ^]^ Although nitrogen accounts for 78% of the volume in the air, the triple bonds of nitrogen molecules with high bond energy (946 kJ mol^−1^) keep high inertness from MOS materials, resulting in almost no resistance variation. Hoa et al. elucidated the chemical inactivity of nitrogen with p‐type CuO thin film.^[^ ^30^ ^]^ Nitrogen is thus commonly used as the carrier gas in the study of chemical kinetics between tested gas and MOS materials.^[^ ^29^ ^]^ Moreover, oxygen accounts for 21% of the volume in the air, which is the second highest concentration gas in the air after nitrogen, and it can be adsorbed on MOS materials to generate enormous influence for gas sensors. Electrons (e^−^) are known as the charge carrier of n‐type semiconductors. When they are exposed to the air, oxygen molecules are adsorbed onto their surface and grab electrons to form active oxyanions, leaving an electronic core–shell configuration with an insulating region above the MOS surface. It is usually called an electron depletion layer (EDL),^[^ ^31^ ^]^ which can increase the electrical resistance of the MOS materials (**Figure** **4**A). The electron distribution in EDL is in a limited depth of Debye length (*λ*_D_, several nanometers) from the surface, which can merely be affected by the adsorption of oxygen molecules.^[^ ^32^ ^]^ Debye length or Debye radius is a parameter about the extent of the net electrostatic effect of charge carriers in solutions, which reflects the charge shielding effect of plasma. The plasma is electrically neutral when the discussed scale is larger than Debye length, otherwise, it is charged. For n‐type semiconductors, Debye length is defined as Equation (5), in which *ε*, *k*_B_, *T*, *e*, and *N*_d_ stand for the relative permittivity, Boltzmann constant, the temperature, elementary charge, and the number density of dopants, respectively.^[^ ^33^ ^]^

(5) $$\lambda_{D}=\sqrt{\frac{\varepsilon k_{B}T}{e^{2}N_{d}}}$$

**Figure 5:**
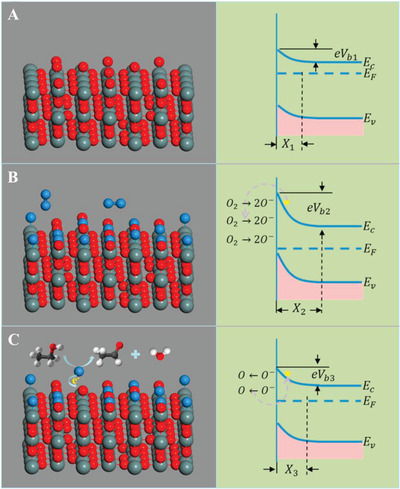
Schematic illustration and the corresponding energy band diagram of A) nonstoichiometric SnO_2_ surface with oxygen vacancies, B) partially repopulated SnO_2_ with adsorbed oxygen, and C) reaction between C_2_H_5_OH and pre‐adsorbed oxygen atoms. Reproduced with permission.^[^ ^35^ ^]^ Copyright 2016, Elsevier.

Li et al. used n‐type SnO_2_ to fabricate a simple but effective sensor for beer detection.^[^ ^35^ ^]^ It elucidated that stoichiometric SnO_2_ was regarded as highly doped semiconductors under an inert or reducing ambiance at high temperatures. This is mainly attributed to the formation of oxygen vacancy sites due to the removal of surface oxygen atoms (Equation (6), **Figure** **5**A). When O_2_ molecules are adsorbed on the surface of MOS materials, they first occupy the previously formed oxygen vacancies and grab electrons from the conduction band of the materials, resulting in the formation of active oxygen species (O_2_^−^, O^−^, and O^2−^) and thus forming an EDL. Generally, O_2_^−^, O^−^ (dissociative oxygen), and O^2−^ (lattice oxygen) become dominant in temperature ranges lower than 150 °C, 150–400 °C, and higher than 400 °C, respectively.^[^ ^31^, ^32^ ^]^ The adsorption and dissociation of oxygen molecules can be further enhanced by increasing operating temperature, doping, and reducing grain size.^[^ ^36^ ^]^ Moreover, when the thickness of the MOS materials is smaller than twice the *λ*_D_, the EDL can be extended to the whole material, resulting in the maximum response. It was experimentally considered in single‐crystal SnO_2_ nanobelts where the EDL has been varied in a few nm‐range by metal catalyst nm‐spots and adsorbed species located at the surface.^[^ ^37^ ^]^ As shown in Figure 5B, the adsorption of oxygen molecules caused the reduction of the Fermi level and the bend of the conduction band to a higher level, which further increased the resistance of the material. For n‐type semiconductors, a redox reaction happens on the surface of MOS materials upon exposing to reducing gases (Figure 5C), reducing the concentration of surface adsorbed oxygen and EDL thickness, and the resistance is thus restored when the Fermi level and conduction band recover to the normal state.

(6) $$O_{0}⇌\ddot{V_{O}}+2e^{\prime }+\frac{1}{2}O_{2}\left(g\right)$$

In terms of the reaction processes, the adsorbed reducing gases are oxidized by various oxygen species and later depleted. At lower temperatures, more active adsorbed oxygen would be prone to react with the reducing gases. However, due to the enhanced surface activities of the sensing materials and depletion of free adsorbed oxygen species at higher temperatures, a transport of oxyanions through the lattice of MOS materials would take place, where lattice oxygen could directly react with the reducing gases and transform into surface oxygen vacancies. Then the surface catalytic activities are further promoted, following a typical MvK mechanism proved by isotope exchange.^[^ ^38^ ^]^ The surface oxygen vacancies can be refilled by curing free oxygen in the air.

**Figure 6:**
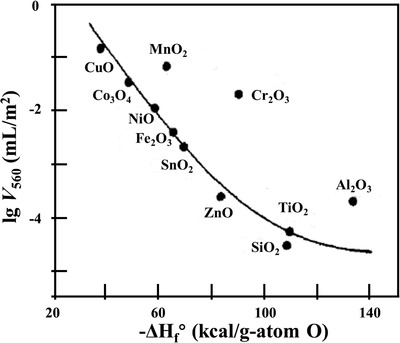
Correlation of the amounts of desorbed oxygen (*V*_560_) with the formation enthalpy of oxides per gram of oxygen (−Δ*H*_f_°). Reproduced with permission.^[^ ^41^ ^]^ Copyright 1978, American Chemical Society.

On the other hand, the holes act as the charge carriers for p‐type semiconductors, and the adsorbed oxygen molecules can reduce the electronic density to form a hole accumulation layer (HAL, Figure 4B).^[^ ^39^ ^]^ It is acknowledged that the electronic core–shell configuration is applicable in both n‐type and p‐type semiconductors, but the core in p‐type semiconductors is insulated while the HAL is conductive, quite different from that of n‐type semiconductors. Therefore, when p‐type semiconductors are exposed to the air, oxygen molecules are first adsorbed onto the oxygen vacancy sites. However, the oxyanions can combine with holes as charge carriers in this case (Equation (7)).^[^ ^40^ ^]^ Iwamoto et al. used the temperature‐programmed desorption (TPD) method to investigate the total amount of oxygen desorption (*V*_560_) on the surface of different MOS materials below 560 °C (**Figure** **6**).^[^ ^41^ ^]^ The p‐type semiconductors such as CuO, Co_3_O_4_, MnO_2_, NiO, and Cr_2_O_3_ tend to show higher *V*_560_ values than the n‐type ones. Moreover, despite some deviations, *V*_560_ values decrease with the increase of formation enthalpy. Generally, increased oxygen adsorption happens on less thermodynamically stable p‐type semiconductors associated with the promotion of redox reactions by variable oxidation states than that on n‐type ones.

(7) $$O_{2}+\ddot{V_{O}}+e^{\prime }⇌{\left(O_{2}^{-}-\ddot{V_{O}}\right)}_{\mathrm{ad}}$$

#### 2.2.2 Chemical Adsorption/Desorption

In general, gas molecules are adsorbed onto the surface of MOS materials, which causes a change in electronic distribution, resulting in a specific resistance variation. Strictly speaking, the above‐mentioned oxygen adsorption on MOS materials is a kind of chemical adsorption. However, due to the existence of oxygen in the air, the oxygen adsorption models should be separately classified and discussed. Although the oxygen adsorption model seems omnipotent in explaining conventional sensing mechanisms of MOS materials, the above theory is inevitably inadequate for various complex situations, in which chemical adsorption and desorption of the tested gas molecules have the main impact on sensing responses.

**Figure 7:**
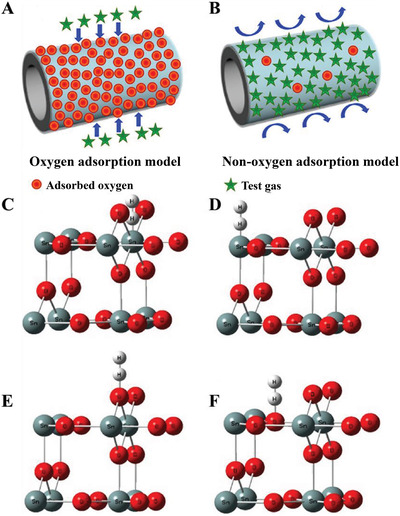
Schematic diagram of gas sensing test: A) oxygen adsorption model, B) non‐oxygen adsorption model. C–F) H_2_ adsorption model of rutile SnO_2_ (110) face (C) H_1_, (D) H_2_, (E) H_3_, and (F) H_4_. Reproduced with permission.^[^ ^45^ ^]^ Copyright 2019, Elsevier.

One of the exceptions to the traditional oxygen adsorption model is oxygen‐free systems. Hahn et al. managed to achieve good responses for CO in low oxygen concentration (20 ppm) by using Pd/Pt‐doped SnO_2_ as sensing materials, and the responses were decreased as oxygen concentration rose.^[^ ^42^ ^]^ The oxygen‐free sensing test for CO is usually carried out under ultra‐high vacuum circumstances, in which the adsorbed CO molecules can react directly with the lattice oxygen (O_lat_) on the surface of the materials, following the MvK mechanism. The number of occupied oxygen vacancies by adsorbed oxygen molecules is low under low oxygen concentrations, and the dominant mechanism is like that of an oxygen‐free mechanism. As the oxygen concentration rises (25–50 ppm), the number of active sites occupied by CO molecules decreases, and the mechanism is transformed into that of the oxygen adsorption model. Further research investigated the impacts of different oxygen backgrounds on H_2_ and CO responses on SnO_2_‐based materials.^[^ ^43^ ^]^ Henrich et al. elucidated a feasible mechanism to explain H_2_ responses under oxygen‐free circumstances,^[^ ^44^ ^]^ including the adsorption of H atoms onto the surface lattice oxygen ions after the dissociation of H_2_ molecules, and the formation of rooted hydroxyl groups (OH_O_^+^), which have lower electronic affinity than oxygen lattice ions and can act as electron donors. Therefore, the number of electrons in the conductance band increases and the resistance decreases. At the same time, an electron accumulation layer (EAL) above the surface is formed to cause the band to bend upwards, which is quite like the HAL in the p‐type semiconductor exposed to the air. However, the increase of conductance with H_2_ concentrations reaches a saturation when the energy level of the electron donor crosses with the Fermi level, due to that the electrons are always under the Fermi level. In short, if sufficient oxygen ions exist on the surface of the materials, the sensing mechanism is dominated by the concentration of surface electron acceptors. However, for materials with a low amount of electron acceptors, surface electron donors dominate the mechanism, resulting in an EAL above an n‐type semiconductor. Both two mechanisms mentioned above happened parallelly under oxygen conditions (**Figure** **7**A, B), but the eventual responses were lower than that of mere hydrogen adsorption in oxygen‐free circumstances. This is because the ionization of electron donors does not increase the concentration of charge carriers on the surface of SnO_2_ under oxygen conditions. Instead, the electrons released by electron donors are eventually trapped by acceptors, resulting from the additional ionosorption of oxygen. No band bending happens due to no change in surface charge. In fact, under oxygen conditions, only the oxygen adsorption model mainly contributes to responses for decreasing concentrations of adsorbed oxyanions and thus increasing concentrations of free charge carriers inside the semiconductors. As a result, the two mechanisms could not be simply overlaid, and similar results were achieved in CO‐sensing experiments.^[^ ^43b^ ^]^ Zhu et al. used Vienna Ab initio Simulation Package (VASP) software to further find out the best adsorption sites as well as relative adsorption energies of H_2_ molecules on the surface of SnO_2_ under oxygen and oxygen‐free circumstances.^[^ ^45^ ^]^ The adsorption energy of oxygen on the most thermodynamically stable (110) surface of SnO_2_ is relatively low (−0.38 eV), which is proved by the first principle calculations, indicating its unstable adsorption. Moreover, the adsorption energy of H_2_ molecules on adsorbed oxygen is even lower (−0.018 eV). On the other hand, among different adsorption sites, the highest adsorption energy of H_2_ molecules (−0.029 eV) is obtained at terminal oxygen sites (Figure 7C–F), suggesting that H_2_ molecules are more likely to be adsorbed directly onto the surface of SnO_2_ under oxygen‐free circumstances rather than on pre‐adsorbed oxyanions, which is consistent with the experimental results. Similarly, some research was carried out upon density‐functional theory (DFT) to derive the adsorption and oxidation mechanisms of CO at different surfaces of SnO_2_ crystals. For example, Lu et al. elucidated that CO oxidation on the SnO_2_ (110) surface follows the MvK mechanism instead of the L–H model, in which the adsorbed oxygen is transformed into various oxyanions by transferring e^−^ to the chemisorbed oxygen.^[^ ^46^ ^]^ Zakaryan et al. further pointed out that the C atoms of CO molecules remained bonded with the lattice oxygen atoms on other surfaces like (101) and (001) of SnO_2_ crystals, leaving the MvK mechanism invalid.^[^ ^47^ ^]^

**Figure 8:**
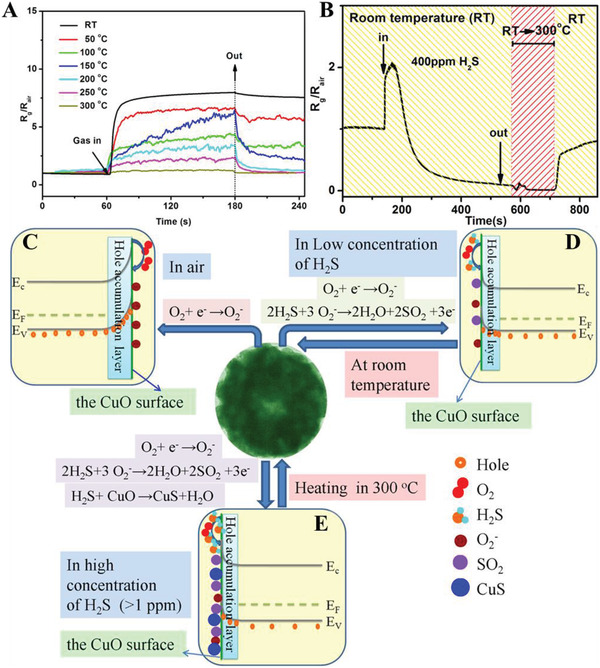
A) Dependence of the CuO sensor's response on the operating temperature in 10 ppm H_2_S; B) gas response of the CuO sensor for 400 ppm H_2_S; C–E) Different sensing mechanisms of the CuO film exposed to different concentrations of H_2_S. (C) In air, O_2_ molecules are adsorbed to form the negative charge oxygen on the CuO surface, which generates the hole‐accumulation layer. (D) When low‐concentration hydrogen sulfide is injected, the electrons released from the reaction of H_2_S with O_2_^−^ adsorbed on the CuO surface decrease the accumulation of holes, thus improving the gas response of the CuO film to H_2_S. (E) When high‐concentration H_2_S is injected, besides oxidation of H_2_S, a CuS layer appears on the CuO surface due to the reaction of H_2_S with CuO, which decreases the gas response of the CuO film to H_2_S. Reproduced with permission.^[^ ^48^ ^]^ Copyright 2019, American Chemical Society.

Another typical situation for a dominant chemical adsorption/desorption is to test the gas molecules which have direct contact with the crystal grains and undergo a chemical reaction, resulting in a change of resistance.^[^ ^28^ ^]^ This process often occurs simultaneously with oxygen adsorption but may contribute much more to the gas responses than the latter. Xu et al. synthesized CuO films composed of monolayer colloidal particles through a self‐assembly procedure, showing a good response for H_2_S gas.^[^ ^48^ ^]^ The reversibility of the CuO sensor was dependent on the concentration of H_2_S. In detail, the H_2_S gas of lower concentration resulted in higher reversibility (**Figure** **8**A), and higher concentration would turn to irreversible response (Figure 8B). As a result, two different mechanisms were elucidated to explain the gas sensing behavior (Figure 8C–E). As a p‐type semiconductor, when CuO is exposed to the air, oxygen adsorption occurs on its surface, resulting in a HAL. Once exposed to H_2_S with a low concentration (< 500 ppb), the gas could be adsorbed and react with existing oxyanions, releasing electrons and nullifying the holes as charge carriers thus increasing resistance (Figure 8C). After pulling out H_2_S and being exposed to the air again, the adsorbed oxyanions are replenished, which can regenerate the nullified holes and recover the resistance.^[^ ^49^ ^]^ Since no sufficient oxyanions occupy the active sites on CuO in the case of higher H_2_S concentration (> 1 ppm), H_2_S molecules tend to be adsorbed and react directly with CuO (Figure 8D).^[^ ^50^ ^]^ As the product CuS has a narrower bandwidth than CuO, the resistance decreases dramatically, which is opposite to the conventional sensing mechanisms for p‐type semiconductors. The sensor cannot completely recover from room temperature to 300 °C, mainly due to the low rate of CuS reoxidation.^[^ ^50^ ^]^ For H_2_S gas in extra high concentration (> 100 ppm), both two mechanisms above contribute eventually to the responses (Figure 8E). As shown in Figure 8B, the oxygen adsorption model dominates the mechanism, and the resistance goes up after injecting H_2_S gas. Even so, the resistance decreases soon due to that chemical adsorption and reaction occur between H_2_S molecules and CuO surface. With more CuS formation, the resistance drops dramatically, indicating that the formation of CuS plays a decisive role in the sensing mechanism. On the other hand, for n‐type MOS sensors, the chemisorption of H_2_S can directly enhance sensor responses. For example, our group has carried out research on the fabrication of H_2_S sensors based on ordered mesoporous MOS materials of Fe_2_O_3_, SnO_2_, and WO_3_.^[^ ^51^ ^]^ The chemisorption of H_2_S molecules onto the MOS sensing layer resulted in the formation of metal sulfides (SnS_2_, WS_2_, etc.), forming a heterojunction with the origin oxide material.^[^ ^51d–f^ ^]^ In this case, the narrower gap width of metal sulfides contributes further to the drop in the resistance in the n‐type sensor with no turnover like that in CuO. However, this can inevitably bring an extended recovery time, as the formed sulfide must be reoxidized before returning to the initial state.

Another common form of chemisorption is the adsorption of water molecules. It is widely acknowledged that the adsorption of water molecules mainly follows the route of physical adsorption, but in practical situations, chemisorption exists as well. The adsorption mechanism of water molecules is determined by the ratio of active sites which are occupied by water molecules. The monolayered chemical adsorption of water molecules is dominant under low humidity, while the multilayered physical adsorption of water molecules is feasible under high humidity due to the occupation of active sites. For gas sensing at low temperatures, humidity harms gas responses as well. In a dry atmosphere with relative humidity (RH) lower than 20%,^[^ ^29^ ^]^ the conventional chemical adsorption/desorption models, especially the adsorption of oxygen molecules mentioned above, play a decisive role in determining the eventual responses. On the other hand, in a humid atmosphere dense water molecules are ionized into H^+^ and OH^−^ ions by dissociative adsorption onto the surface.

**Figure 9:**
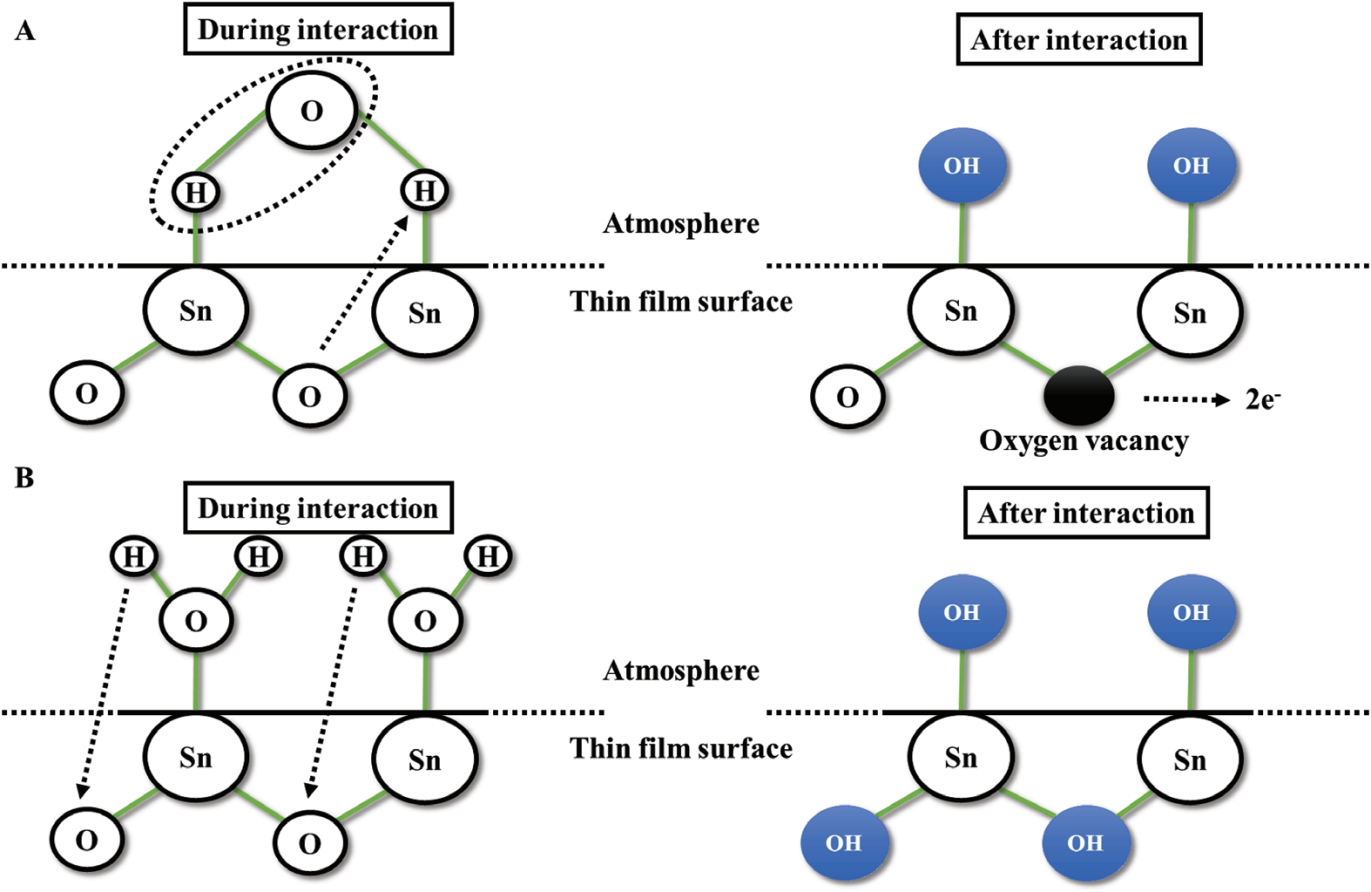
Mechanisms of humidity adsorption on the surface of tin oxide: A) one water molecule for two metal sites and B) one water molecule per metal site. Reproduced with permission.^[^ ^29^ ^]^ Copyright 2015, Cognizure.

Heiland et al. elucidated two different adsorption mechanisms of water molecules on SnO_2_ surfaces.^[^ ^52^ ^]^ In the case that one water molecule reacts with two metal sites (Equation (8), **Figure** **9**A), the dissociated OH^−^ ions form chemical bonds directly with Sn atoms, and lattice oxygen combines with the left H^+^ ions to form two Sn—OH dipoles and release two free electrons. On the other hand, in the reaction between one water molecule and one metal site (Equation (9), Figure 9B), the ionized H^+^ atoms diffuse into deeper areas of the material and then combine with the lattice oxygens, where the remaining OH^−^ ions also combine with the Sn sites. Since the rooted OH groups show low electronic affinity and ionization degrees, they act as electron donors. In short, the adsorption of water molecules causes a change in gas responses, which may result in a change in conductivity or the occupation of active sites. These theories are far from being adequate to omnipotently explain all mechanisms including the adsorption of water molecules because most of them neglect the physical adsorption of water molecules, which will be discussed in the following part.

(8) $$H_{2}O\left(g\right)+2\left({\mathrm{Sn}}_{\mathrm{Sn}}+O_{O}\right)⇌2\left({\mathrm{Sn}}_{\mathrm{Sn}}^{\delta +}-{\mathrm{OH}}^{\delta -}\right)+V_{O}^{2+}+2e^{-}$$

(9) $$H_{2}O\left(g\right)+\left({\mathrm{Sn}}_{\mathrm{Sn}}+O_{O}\right)⇌\left({\mathrm{Sn}}_{\mathrm{Sn}}^{\delta +}-{\mathrm{OH}}^{\delta -}\right)+{\left(\mathrm{OH}\right)}_{O}^{+}+e^{-}$$

In a word, chemical adsorption/desorption, which is closely relevant to the gas sensing process, is often overlooked in most reports. Therefore, the chemical adsorption/desorption model is an important supplement for conventional oxygen adsorption.

#### 2.2.3 Physical Adsorption/Desorption and Humidity Effects

Physical adsorption is another common adsorption process in sensing. However, unlike the oxygen adsorption model and chemical adsorption mentioned above, the theory of physical adsorption is seldomly applied in explaining gas sensing mechanisms, because the change of conductance caused by physical adsorption is negligible under most circumstances.^[^ ^28^ ^]^ As mentioned above, physical adsorption dominates the primary adsorption forms of water molecules. Therefore, the humidity sensor is developed as one of the most common MOS sensors based on the physical adsorption mechanism. Even so, the response of the humidity sensors is still influenced by chemical adsorption including oxygen adsorption.^[^ ^53^ ^]^

**Figure 10:**
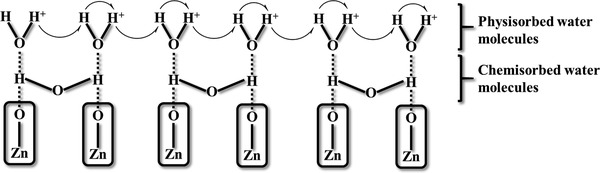
Schematic representation of humidity sensing at ZnO. Reproduced with permission.^[^ ^58^ ^]^ Copyright 2010, Elsevier.

**Figure 11:**
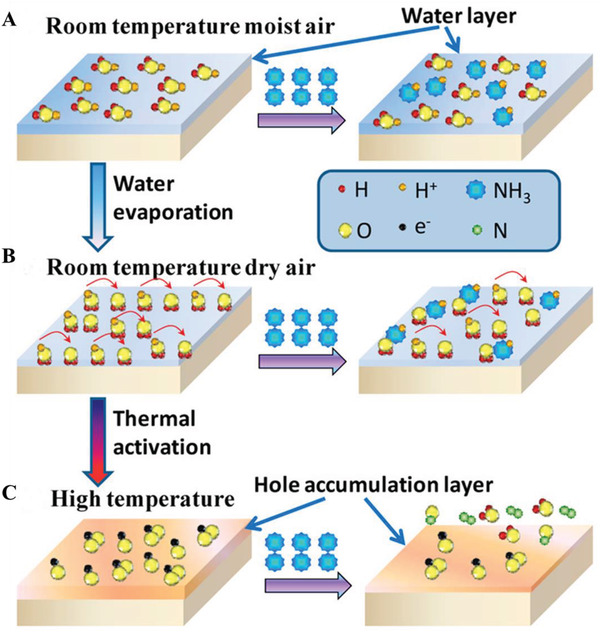
Schematic diagram of the sensing mechanism of the CuScO_2_ at different operating conditions: A) at RT in moist air, B) at RT in dry air, and C) at high temperature. Reproduced with permission.^[^ ^56^ ^]^ Copyright 2019, American Chemical Society.

Besides chemical adsorption in low humidity discussed above, physical adsorption in higher humidity accounts for a lot in the adsorption mechanism of water molecules. Morrison further suggested the concept of co‐adsorption under even more humid circumstances.^[^ ^54^ ^]^ Water molecules expel other molecules that have already occupied the active sites, and both monolayered chemical adsorption and multilayered physical adsorption happen simultaneously to cause co‐adsorption. In this case, the H^+^ ions in the physical adsorption layer can flow freely among water molecules by hydrogen bonds according to the Grotthuss proton hopping mechanism (**Figure** **10**).^[^ ^55^ ^]^ Specifically, due to the close relationship between adsorption and diffusion, the detailed hopping mechanism will be discussed in **Section** **3**. The resistance change varies in different adsorption forms, and generally, chemisorption is the highest, physisorption is moderate and co‐adsorption is almost zero. In some cases, humidity has an impact on the intrinsic nature of the materials as well. For example, Deng et al. prepared p‐type CuScO_2_ sensors toward NH_3_, showing pseudo‐n‐type properties.^[^ ^56^ ^]^ Due to the highly resistive nature of CuScO_2_, the resistance in humid conditions at room temperature is mainly dominated by the surface adsorbed water layers, in which NH_3_ molecules are dissolved and ionized to contribute NH_4_^+^ and OH^−^ ions to the water layer. In addition, the free flow of H^+^ ions contributes to the conductance of the material following the Grotthuss proton hopping mechanism, thus a pseudo‐n mechanism is established. After that, due to the higher proton affinity of NH_3_ than that of water molecules,^[^ ^57^ ^]^ the moving protons can be trapped by NH_3_ molecules, following a pseudo‐p mechanism (**Figure** **11**A). The desorption of water molecules weakens the pseudo‐p responses to water evaporation (Figure 11B). All water molecules are desorbed at higher temperatures (> 100 °C), leaving intrinsic p‐type responses for CuScO_2_ (Figure 11C).

Besides the adsorption of water molecules, physical adsorption of oxygen molecules occurs at room temperature as well. Hong et al. used a field‐effect transistor (FET)‐type oxygen gas sensor to observe the physical adsorption of oxygen molecules at room temperature, which was thought to have little impact on gas responses in most cases.^[^ ^59^ ^]^ However, even in the oxygen adsorption model, the physical adsorption of oxygen molecules on the surface of materials at low temperatures is dominant. Although such physical adsorption has no direct electronic effect, it is estimated to interfere with the direct transport of charge carriers at the surface of the materials, resulting in a slight increase of resistance via mobility reduction. Primarily, this effect seems to dominate in low‐noised (quasi‐) 2D materials like graphene or Mxene layers in difference to bulk, even mesoporous, metal oxides where the chemiresistive matures mostly from variations in the free carrier concentration under chemisorption.^[^ ^60^ ^]^

#### 2.2.4 Theory of Power Law for MOS Gas Sensors

The correlation between the resistance of a MOS gas sensor and gas concentration has been studied for a long time. In 1987, Morrison elucidated a mass action law for reducing gases on SnO_2_ materials that the resistance varied as *P*_R_^−0.5^ at low *P*_R_, in which *P*_R_ was the partial pressure of the reducing gas.^[^ ^61^ ^]^ Some empirical results were acknowledged from then on,^[^ ^9^, ^62^ ^]^ but a systematic theory explaining the origination of power law still lacked. Later, based on the theories of gas adsorption mentioned above, Yamazoe et al. elucidated a theoretical basis for the power law theory of MOS gas sensors,^[^ ^10g^ ^],^ that is, the resistance of a MOS material exposed to a kind of gas with a partial pressure *P* is proportional to *P^n^*, in which *n* remains a constant to a specific target gas. Conventionally, the resistance was thought to have a roughly linear correlation with gas concentration, especially under low concentration circumstances, which was further applied in diffusion models,^[^ ^10c^ ^]^ calculation for the limit of detection (LoD),^[^ ^63^ ^]^ etc. Moreover, the power law can be promoted to extensive research on gas diffusion.^[^ ^61^, ^64^ ^]^ The power law can be achieved by the combination of donor function (adsorption and reaction of gas molecules) and transducer function (change in surface potential), that is, the combination of chemical adsorption on the oxide surface and physical adsorption on the MOS surface. Some basic theories like that of conventional semiconductors and grain impacts (double Schottky layer and tunneling model) were applied.^[^ ^32^, ^65^ ^]^ The chemical change after the adsorption of different gas molecules was further discussed. For simplification, the oxyanion formed after oxygen adsorption was assumed to be O^−^. The accumulation of O^−^ can be expressed by Equation (10), in which *k*_1_ and *k*_‐1_ stand for rate constants of the forward and reverse reactions, respectively. On the other hand, the density of conduction electrons in the physical change on the surface of an n‐type semiconductor can be defined as Equation (11), in which *N*_d_ and *m* stand for the number density of electron donors and reduced depletion depth, respectively. By combining the chemical change and physical change mentioned above, the power law exponent *n* can be eventually expressed in Equation (12). When *m* is sufficiently large, *n* equals 0.5, which is a limit in the power law. Yamazoe et al. later extended the power law derivation to other oxyanion species and elucidated that the power law exponent *n* is 0.25 if the adsorbed oxyanion is O^2−^.^[^ ^66^ ^]^

(10) $$\frac{d[O^{-}]}{dt}=k_{1}Po_{2}{\left[e^{-}\right]}^{2}-k_{-1}{\left[O^{-}\right]}^{2}$$

(11) $$\left[e^{-}\right]=N_{d}e^{-\frac{m^{2}}{2}}$$

(12) $$n=\frac{\mathrm{dlg}R}{\mathrm{dlg}Po_{2}}=\frac{m^{2}}{2(m^{2}+1)}$$

**Figure 12:**
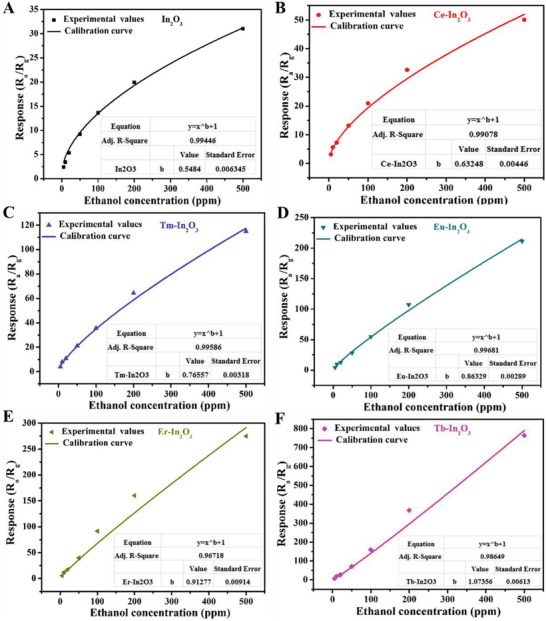
Calibration curves of response versus ethanol concentration of pristine and RE‐doped In_2_O_3_ NTs in the range of 5–500 ppm. Reproduced with permission.^[^ ^71^ ^]^ Copyright 2020, Elsevier.

As mentioned above, the oxyanion species formed on the surface of MOS sensing materials differ with operating temperatures, and so does the power law exponent. Practically, the sensing response of a gas sensor is commonly defined as *S* = *R*_a_/*R*_g_ or *S* = (*R*_a_−*R*_g_)/*R*_a_ × 100%. In particular, a redox interaction at the surface of SnO_2_ can explain the chemiresistive response *S*, as primarily suggested by Windischman et al. for the exemplary case of CO adsorption (Equation (13)),^[^ ^67^ ^]^ in which *e*, *µ*, *d*, *α*, *γ*, and *P*_R_ stand for the elementary charge, electron mobility, layer thickness or characteristic grain size, the coefficient characterizing a sticking of reducing gas R to the surface, surface recombination coefficient, and partial pressure of R gas, respectively. According to numerous experimental results where simple gas molecules interact with the oxide surface and single adsorbed species yield one free electron (to the conductance band of the material), the power law index *β* in Equation (13) equals 0.5. In the framework of this approach, when larger gaseous molecules, such as volatile organic compounds (VOCs), are considered, the power law index deviates from the 0.5 value due to appearing more than one electron as a sequence of surface reactions. Besides the change in gas molecules, Scott et al. investigated the impact of MOS surface modification on *β* values, in which SnO_2_ inverted opals acted as sphere agglomerates.^[^ ^68^ ^]^ The expected *β* value may shift from 0.5 to values smaller than 1 for disordered microstructures or local agglomeration,^[^ ^69^ ^]^ depending on the charge state of surface oxyanion species.^[^ ^68^, ^70^ ^]^ Specifically, the high surface coverage of O^2−^ species leads to *β* values lower than 0.5, and abundant O^−^ species may help shift the value near 1 and boost sensing responses.^[^ ^71^ ^]^ Bai et al. synthesized a series of ethanol sensors by doping different rare earth (RE) elements on In_2_O_3_ nanotubes (NTs) with abundant oxygen vacancies,^[^ ^71^ ^]^ and the power laws between gas response and sensors doped with different rare earth elements were further investigated. The *β* value for pure In_2_O_3_ NT with no doping was quite close to 0.5 (**Figure** **12**A), consistent with the previous models. After doping different RE elements on In_2_O_3_ NT, the *β* value slightly shifted higher than 0.5, together with a rise in the proportion of O^−^ species among different oxyanions due to the increase of surface oxygen vacancies, leading to the enhancement of sensing responses. An ultra‐high *β* value of 1.07356 for Tb‐In_2_O_3_ exceeded the conventional extreme value of 1 (Figure 12F), which mainly resulted from the extra‐high proportion of O^−^ species on the material surface.

(13) $$S=e\mu d{\left(\frac{\alpha }{\gamma }P_{R}\right)}^{\beta }$$

It should be noted that the expansion of linear correlation into power law is mainly critical under circumstances with high gas concentrations. Generally, high‐concentration gas testing usually occurs when practical applications are significant (e.g., H_2_),^[^ ^72^ ^]^ or the test gas itself is too stable to achieve decent responses at low concentrations (e.g., CH_4_).^[^ ^73^ ^]^ On the other hand, under most circumstances, the linear correlation is adequate for the conventional concentration range tested. For example, the concepts of LoD and limit of quantitation (LoQ) require an extremely low concentration at which the response is comparable to background noise.^[^ ^74^ ^]^ In this case, the power law is surely reduced to linear correlation in practical calculations.^[^ ^63^ ^]^

#### 2.2.5 Impacts of Gas Adsorption/Desorption on Response/Recovery Processes

The response of a MOS sensor is derived from the change of resistance or conductivity when switching to a kind of test gas. The resistance returns to the original value when switching back to the air. Conventionally, if the conductivity of a sensing material increases on a kind of gas, for example, an n‐type semiconductor is exposed to a kind of reducing gas, its response is defined as *S* = *R*_a_/*R*_g_ or *S* = (*R*_a_−*R*_g_)/*R*_g_ × 100%, in which *R*_a_ and *R*_g_ stand for the resistance of the material exposed to the air and the test gas, respectively. On the other hand, if the conductivity decreases, the response is defined as *S* = *R*_g_/*R*_a_ or *S* = (*R*_g_−*R*_a_)/*R*_a_ × 100%. Empirically, the value of response/recovery is measured by the time (*τ*) needed for a 90% full response/recovery.^[^ ^8^ ^]^ In practical experiments, since the response of a sensor is measured under the two equilibrium states, the intermediate response/recovery transients are usually overlooked, in which the dynamic measurement must be adopted.^[^ ^75^ ^]^ The response transient of the sensor is probably determined by the chemistry on and in the sensing layer,^[^ ^10a^ ^]^ which has a significant role in the gas sensor.

**Figure 13:**
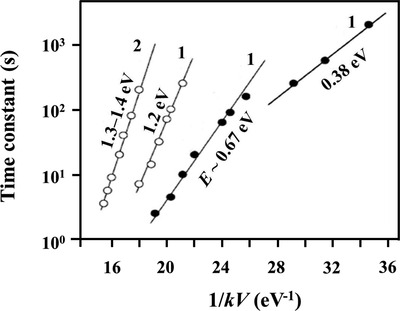
Temperature dependencies of time constants of transient characteristics of SnO_2_ film gas response, measured in wet atmosphere (30–50% RH): 1) *d* ≈ 30–60 nm; 2) *d* ≈ 200 nm. Reproduced with permission.^[^ ^10f^ ^]^ Copyright 2004, Elsevier.

Lundström discussed the response/recovery transients in solid sensing materials.^[^ ^10a^ ^]^ By applying a Langmuir‐like isotherm and assuming first‐order dynamics, two‐time constants (*τ*_f_ and *τ*_r_) are introduced, which represent forward and reverse reactions for the response and recovery processes, respectively. The initial rates of response and recovery are further calculated as Equations (14) and (15), in which *Θ* stands for the response. The value of Equation (14) is always larger than Equation (15), indicating that in the first‐order dynamics, recovery time is longer than response time. Further derivation was carried out on the second‐order dynamics (e.g., the adsorption of H_2_ on Pd,^[^ ^76^ ^]^ in which PdH*_x_* is formed), and the response and recovery rates were calculated to be comparable. Korotcenkov et al. discussed the response/recovery dynamics in SnO_2_ sensing.^[^ ^10f^ ^]^ Several different ranges of working temperatures and relative activation energies are measured (**Figure** **13**). Further experiments revealed that lattice oxygen had no evident impact on the formation of water molecules, therefore, under oxygen‐free circumstances, the response dynamics were determined by the material itself, and in oxygen situations, the distinction of response and recovery time was eliminated. Furthermore, response time is comparable to or longer than recovery time at above 150 °C, while below 150 °C response time is shorter than recovery time. The former belongs to the first‐order dynamics, of which the rate‐limiting step is the adsorption/desorption process with no further dissociation. While the latter belongs to the second‐order dynamics, where the rate‐limiting step is the dissociative adsorption of surface substances, especially OH— groups and oxygen atoms.

(14) $${\left(\frac{d\Theta }{dt}\right)}_{0}=\frac{1}{\tau_{f}}$$

(15) $${\left(\frac{d\Theta }{dt}\right)}_{0}=\frac{1}{\tau_{f}+\tau_{r}}$$

Further research was carried out on the dynamics of In_2_O_3_ sensors.^[^ ^77^ ^]^ The transient change in SnO_2_ mainly has a power correlation with time,^[^ ^10f^ ^]^ whereas In_2_O_3_ has more complicated relations such as power, square root, and direct proportion, resulting from large amounts of adsorption sites for different oxygen species like chemisorbed oxygen, oxygen vacancies and lattice oxygen. In_2_O_3_ showed different behaviors when exposed to different gases at different temperatures. For CO and H_2_, an acceptor behavior was observed below 250 °C, with a donor behavior instead above 250 °C. While for ozone, the time constant of the recovery process (*τ*_rec_) is far longer than that of the response process (*τ*_res_). The mechanism of response and recovery processes for In_2_O_3_ was further elucidated, which could be mainly divided into two different processes, the adsorption/desorption process, and the redox process. The former process mainly consists of adsorption/desorption and dissociation of gas molecules, surface oxygen diffusion, surface reaction, and desorption of the products. Apart from that, in the latter process, the interaction between gas molecules (oxygen, water, and test gas) and In_2_O_3_ lattice (reduction or reoxidation), surface construction, and bulk diffusion of oxygen species or oxygen vacancies must be considered as well. For example, the recovery process for CO and H_2_ is the surface reoxidation process of In_2_O_3_, but for ozone that is merely desorption. In the redox process, response time is overall comparable with recovery time, while in the adsorption/desorption process recovery time is longer. Moreover, although reducing gases mainly follow the redox process, the adsorption/desorption of oxygen molecules is also a significant part. For example, the oxidation reaction between CO and different oxygen species has different results, in which lattice oxygen mainly acts as a donor, while adsorbed oxygen acts as an acceptor, and response time upon adsorbed oxygen is shorter than that with lattice oxygen. Besides pure MOS materials, more research has been carried out on complex materials, such as polymers and heterojunction materials.^[^ ^78^ ^]^ For example, Hu et al. analyzed the adsorption dynamics of NH_3_ on polyaniline thin films, in which both Langmuir and Freundlich models were applied, and the response of NH_3_ was found to mainly obey the latter process.^[^ ^78a^ ^]^ On the other hand, it is a common misconception to choose the model based on the best fitting data,^[^ ^14^ ^]^ so its validity might be challenged.

**Figure 14:**
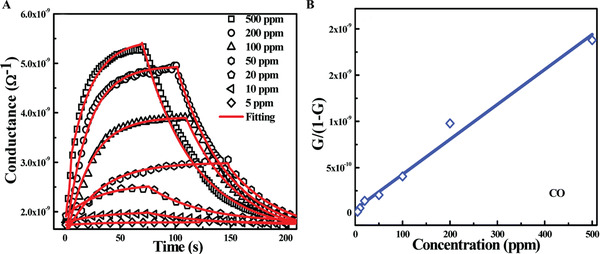
A) Response and recovery transients of Co‐doped ZnO thin film sensors (symbols) exposed to various CO concentrations (ranging from 5 to 500 ppm) measured at 300 °C, and the experimental data were fitted (solid lines) for response and recovery; B) linear plot of *G*/(1−*G*) versus CO concentration. Reproduced with permission.^[^ ^64^ ^]^ Copyright 2017, Royal Society of Chemistry.

**Figure 15:**
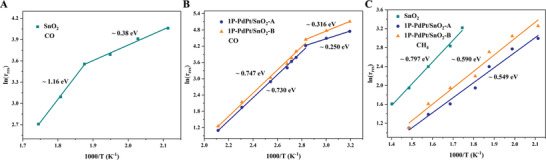
Temperature dependence of linear fitted plots of ln(*τ*_res_) in the presence of A,B) CO, C) CH_4_ using SnO_2_, 1P‐PdPt/SnO_2_‐A, and 1P‐PdPt/SnO_2_‐B as sensing materials. Reproduced with permission.^[^ ^73^ ^]^ Copyright 2019, American Chemical Society.

Mukherjee et al. further used zinc ferrite sensors to determine the response and recovery dynamics of H_2_.^[^ ^75^ ^]^ Taking the first‐order dynamics into account, it is confirmed that the reaction between chemisorbed oxygen and tested gas is the rate‐limiting step for the response process, and the desorption of oxidized product is the rate‐limiting step for the recovery process.^[^ ^64^ ^]^ On the other hand, the temperature dependences of time constants follow the Arrhenius equation (Equations (16) and (17)), in which *E*_A_ and *E*_D_ stand for the activation energy for test gas adsorption and reaction product desorption, respectively, and *τ*_0_ and *τ*_0_’ stand for pre‐exponential constants, which are determined only by the reaction nature.^[^ ^73^ ^]^ Based on this consideration, Ghosh et al. further carried out CO and H_2_ sensing experiments on Co‐doped ZnO.^[^ ^64^ ^]^ As shown in **Figure** **14**A, both *G*_0_ and *G*_0_’ remained almost invariant to CO concentration, and the linear correlation between *G*/(1−*G*) and CO concentration (Figure 14B) indicated a Langmuir adsorption isotherm.^[^ ^78a^ ^]^ Li et al. synthesized PdPt alloy‐doped SnO_2_ nanosheets that showed a dual selectivity toward CO and CH_4_ at different temperatures.^[^ ^73^ ^]^ The dynamic response processes were also investigated (**Figure** **15**). For relatively active CO molecules, the dynamic processes showed a turning point (Figure 15A, B), in which activation energies at higher temperatures are higher than that at lower temperatures, indicating the existence of two different paths for CO oxidation. At lower temperatures only surface‐adsorbed oxygen participates in the oxidation, and at higher temperatures both surface‐adsorbed oxygen and lattice oxygen participate, thus increasing mean activation energies. As CH_4_ molecules have quite stable tetrahedral structures (Figure 15C) which need higher temperatures for activation, only one route including both surface‐adsorbed oxygen and lattice oxygen is observed. Moreover, PdPt alloy doped SnO_2_ (denoted as 1P‐PdPt/SnO_2_‐A) displayed the lowest activation energies (Figure 15, blue lines) among different sensing materials, mainly resulting from the spill‐over effect, in which the noble metals acted as active sites and the dissociative adsorption of oxygen was strengthened around them to improve gas oxidation.^[^ ^36^, ^79^ ^]^

(16) $$\tau_{\mathrm{res}}=\tau_{0}e^{\frac{E_{A}}{2k_{B}T}}$$

(17) $$\tau_{\mathrm{rec}}=\tau_{0}^{\prime }e^{\frac{E_{D}}{2k_{B}T}}$$

## 3 Gas Diffusion

### 3.1 Classical Diffusion Theories and Models

#### 3.1.1 Fick's Laws of Diffusion

In thermodynamics, transport means the process in which the thermodynamic system undergoes a transition from the non‐equilibrium state to the equilibrium one, such as heat conduction, viscosity, and diffusion. When the density of particles in the system is not uniform, they will migrate from the places with higher concentrations to that with lower ones through thermal motion, and this process is called diffusion. Under actual circumstances, the diffusion processes always go along with other macro motions in the system, which makes diffusion itself quite complicated. Specifically, in a one‐species system with uniform temperature and pressure, the pure diffusion merely originating from concentration difference is called self‐diffusion, and the process in the systems containing different particles with similar diffusivity (e.g., CO and N_2_) is called interdiffusion. In 1855, A. Fick developed two laws about the innate character of diffusion. The first law relates the diffusive flux with the gradient of concentration, indicating that the flux goes from higher concentration regions to lower ones. Fick's first law can be written in various forms based on different environments, and Equation (18) is most used under 1D circumstances, in which *J* stands for the diffusive flux, which measures the amount of substance undergoing the diffusion process; *D* stands for the diffusion coefficient or diffusivity; *φ* stands for the concentration of substances and *x* stands for the position, that is, the length along the 1D diffusion route. Fick's second law predicts how diffusion affects concentration change along with time. Equation (19) is the most used, in which *t* stands for time. Fick's second law can be further derived from Fick's first law and the law of energy conservation.

(18) $$J=-D\frac{d\varphi }{dx}$$

(19) $$\frac{\partial \varphi }{\partial t}=D\frac{\partial^{2}\varphi }{\partial x^{2}}$$

#### 3.1.2 Hard‐Sphere Model and Derivation of the Diffusion Coefficient

Based on the collision theory and Newton's first law, a hard‐sphere model is widely applied in the simulation of molecular dynamics (MD).^[^ ^80^ ^]^ Therefore, if a molecule is simplified as a hard sphere with a diameter of *d* (molecular kinetic diameter),^[^ ^81^ ^]^ it performs a uniform linear motion in space before colliding with another one. The whole route of the molecule is a broken line, and thus the collision range of a molecule in space can be simplified as a zigzag cylinder with an underside radius of *d*. The length of the route that a molecule goes along the zigzag cylinder before a collision is defined as its free path (*λ*). The mean free path of a certain kind of gas molecule can be calculated according to Maxwell's distribution law (Equation (20)), in which $\bar{\lambda }$, *n*, and *σ* stand for the mean free path of molecules, the number density, and effective collision area, respectively. For ideal gases, Equation (20) can be expressed as Equation (21), in which *k*_B_, *T*, and *p* stand for Boltzmann constant, temperature, and pressure, respectively. The correlation between mean free path $\bar{\lambda }$and temperature *T* is a critical factor, and it seems that $\bar{\lambda }$ is proportional to *T* according to Equation (21). However, the parameter *T* does not appear in Equation (20) and thus $\bar{\lambda }$ is thought to be independent of temperature at a constant volume. In fact, as temperature rises, the average traveling distance before collision remains unchanged, although the mean collision time decreases. On the other hand, $\bar{\lambda }$ slightly decreases as temperature increases. This phenomenon mainly results from the strengthening of molecular vibration during temperature increase, causing the *σ* value to slightly increase and thus decreasing the mean free path. The manipulation of the mean free path by adjusting the temperature is a key method in improving gas responses,^[^ ^82^ ^]^ which will be discussed later. The diffusion coefficient (*D*) can be further defined as Equation (22),^[^ ^80b^ ^]^ in which ${\bar{u}}_{A}$, *R*, and *M* stand for the root‐mean‐square (RMS) velocity of the gas molecules, the universal gas constant, and molar weight, respectively.^[^ ^83^ ^]^ For ideal gases, Equation (23) can be further expressed, in which *N*_A_ stands for Avogadro constant. As *D* is inversely proportional to *M* ^0.5^ as well as *d* ^2^, gas molecules with smaller molar weights and kinetic diameters are prone to diffuse, leaving larger ones behind.^[^ ^81^ ^]^

(20) $$\bar{\lambda }=\frac{1}{\sqrt{2}n\sigma }$$

(21) $$\bar{\lambda }=\frac{k_{B}T}{\sqrt{2}p\sigma }$$

(22) $$D=\frac{1}{3}{\bar{u}}_{A}\bar{\lambda }=\frac{1}{3}\bar{\lambda }\sqrt{\frac{8\mathrm{RT}}{\pi M}}$$

(23) $$D=\frac{2}{3\sqrt{M}d^{2}pN_{A}}{\left(\frac{\mathrm{RT}}{\pi }\right)}^{\frac{3}{2}}$$

#### 3.1.3 Confined Diffusion and Diffusion Mechanisms in Porous Materials

**Figure 16:**
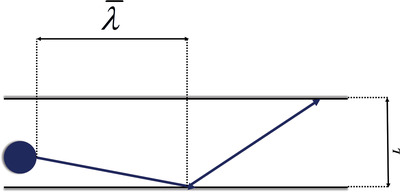
Schematic illustration of the mean free path of a particle and its physical length scale of diffusion.

Apart from the normal Fickian diffusion, in which gas molecules diffuse freely and collide with each other,^[^ ^84^ ^]^ the confined diffusion usually happens within an external physical scale (e.g., size of the gas container or diameter/radius of pores in porous materials, **Figure** **16**).^[^ ^85^ ^]^ In fluid mechanics, the relationship between mean free path and physical length scale is determined by a dimensionless parameter named Knudsen number (*Kn*), which is defined in Equation (24), where *L* stands for representative physical length scale of diffusion. A higher Knudsen number indicates a larger mean free path of fluid than the physical length scale, thus encouraging fluid to pass more freely through the representative container. Typically, according to different values of *Kn*, four kinds of different flows are empirically defined.^[^ ^86^ ^]^ *Kn* < 0.01—continuum flow or Poiseuille flow—the mean free path of fluid is much smaller than the physical length scale, and thus fluid can pass through the pores in the form of continuous mass (bulk fluid flow) rather than discrete particles.^[^ ^86^ ^]^ For macro systems, the fluid concentration is quite high, which thus causes a rise in viscosity. *Kn* > 10—free‐molecular flow or Knudsen flow—the flow of particles can be independent of each other, and all of them move along straight lines.^[^ ^86^ ^]^ For macro systems, the fluid concentration is very low (usually below 0.1 Pa), and the fluid is in a high vacuum or ultra‐high vacuum state, thus the viscosity is almost zero. 0.01 < *Kn* < 0.1—slip flow—fluid particles move in the form of smaller masses than that in continuum flow, but they still have a connection with each other.^[^ ^86^ ^]^ For macro systems, the fluid pressure is relatively high. 0.1 < *Kn* < 10—transitional flow—the mean free path is comparable to the representative physical length scale, and it is the superposition of continuum flow and free‐molecular flow. The fluid shows the properties of continuum flow and free‐molecular flow at the same time.^[^ ^86^ ^]^ Although flow and diffusion are similar and happen simultaneously in some systems, they are driven by different aspects. The former is driven by a pressure gradient, while the latter is driven by a concentration gradient. In other words, flow and diffusion cannot happen in systems in which particles and molecules are uniformly distributed with no gradient‐driven mass transfer.

(24) $$Kn=\frac{\bar{\lambda }}{L}$$

**Figure 17:**
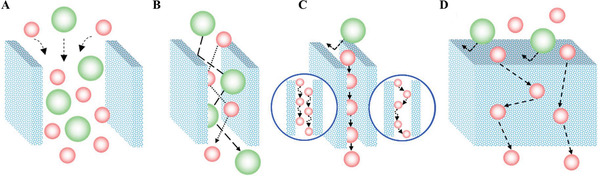
Schematic diagrams of gas diffusion mechanisms A) bulk Poiseuille flow, B) Knudsen diffusion, C) size‐restricted diffusion (left inset: surface model and right inset: gas‐translational model), and D) solid‐state diffusion mechanism. Reproduced with permission.^[^ ^87^ ^]^ Copyright 2017, Elsevier.

The gas molecules first diffuse into sensing materials and then react on active sites. In porous materials, different pore sizes cause a change in *Kn* values, thus producing different forms of diffusion. Li et al. studied the particle diffusions in membranes and illustrated different gas diffusion mechanisms in membranes with different pore sizes, as shown in **Figure** **17**.^[^ ^87^ ^]^ Bulk Poiseuille flow (Figure 17A) or convective flow happens when the pore size is larger than $\bar{\lambda }$. In this case, different gas molecules can freely pass through the pores in the form of bulk fluid flow thus causing quite a low selectivity. Knudsen diffusion (Figure 17B) occurs when the scale of the diffusion system is comparable to or smaller than $\bar{\lambda }$, thus *Kn* value is comparable to or a bit larger than 1. In this case, the gas molecules may collide with the sensing materials (membrane, MOS materials, etc.) during the diffusion process. The pore radius *r* of porous materials can be denoted as the diffusion scale ($\bar{\lambda }/2$), and thus, the Knudsen diffusion coefficient (*D*_K_) can be expressed in Equation (25), combining the intrinsic molecular parameters (*M*) with the extrinsic diffusion scale (*r*). Similarly, the inversely proportional correlation between *D*_K_ and the square root of *M* enables Knudsen diffusion to display a little higher selectivity than bulk Poiseuille flow, but it is still not sufficient. Size‐restricted diffusion (Figure 17C) or molecular sieve diffusion happens when the pore size is between two different gas molecules, and thus larger molecules (green ball) are not able to diffuse into the pores, leaving only smaller molecules (red ball) to pass through the pores, resulting in higher selectivity. The size‐restricted diffusion can be further divided into a surface model (Figure 17C, left inset) and a gas translational model (Figure 17C, right inset).^[^ ^87^ ^]^ Solid‐state diffusion (Figure 17D) is a special kind of membrane diffusion, and there are almost no membrane pores. In this case, gas molecules are adsorbed onto the membrane surface and some of them are dissolved into the membrane materials. After a series of processes inside membrane materials, the gas molecules are desorbed from the other side of the membrane surface and then released into space.

(25) $$D_{K}=\frac{4r}{3}\sqrt{\frac{2RT}{\pi M}}$$

**Figure 18:**
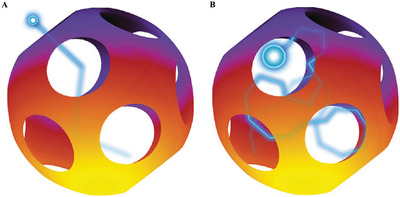
Sketch of the process of multiple collisions that a tracer particle undergoes against the cavity walls before it escapes to the neighboring cavity. A) For small ratios *a*/*L* between particle size and cavity openings, a small number of bounces suffices, B) but for large ones, a large number of collisions with the walls takes place before the particle escapes. Reproduced with permission.^[^ ^84^ ^]^ Copyright 2011, American Chemical Society.

It should be noted that the above four diffusion mechanisms are mainly appropriate in membrane diffusion, however, there are fewer kinds of diffusion mechanisms for other materials like porous MOS materials, in which the range of manipulated pore sizes is not as wide as that in membranes. Specifically, if the pore size is too small for gas molecules to pass, resulting in 1D channels, size‐restricted diffusion happens, which is called single‐file diffusion or configurational diffusion.^[^ ^88^ ^]^ Some other concepts for the confined diffusion in porous materials have been elucidated as well (e.g., incommensurate diffusion and levitation effects),^[^ ^89^ ^]^ but their intrinsic mechanisms remain unclear.^[^ ^90^ ^]^ Another empirical classification of different diffusion modes contains molecular, Knudsen, configurational, and surface diffusion.^[^ ^88a^ ^]^ According to the classification of IUPAC, pores with a size lower than 2 nm are defined as micropores, and those larger than 50 nm are macropores, while those with a pore size between 2–50 nm are mesopores. The diffusivity of the gas molecules into pores rises as pore sizes go up,^[^ ^10c^ ^]^ and thus they undergo the process from surface diffusion and Knudsen diffusion to bulk molecular diffusion.^[^ ^49^, ^91^ ^]^ When the value of mean free path ($\bar{\lambda }$) is comparable with pore sizes of 1–100 nm, the Knudsen diffusion becomes dominant. In other words, larger pores can encourage gas molecules to freely diffuse, and the confinement of diffusion is more significant in smaller pores.^[^ ^33^ ^]^ Therefore, the wide usage of mesoporous materials in gas sensing not only results from their intrinsic advantages,^[^ ^51^, ^92^ ^]^ but also contributes to the Knudsen diffusion of gas molecules,^[^ ^33^, ^51^, ^92^, ^93^ ^]^ which can further boost the sensing abilities. Some researchers have carried out investigations on distinguishing Fickian and confined diffusion in porous materials. Raccis et al. used Brownian dynamics (BD) simulations on inverse opal models (**Figure** **18**) with various cavity sizes and openings by tuning the parameters of particle radius (*a*), hole diameter (*L*), and cavity radius (*R*).^[^ ^84^ ^]^ Normal Fickian diffusion is predicted under all circumstances, while non‐Fickian exponents are attributed to cavity‐opening polydispersity. Malek et al. investigated the impacts of surface roughness of nanoporous media on Knudsen self‐diffusion and Fickian diffusion by dynamic Monte Carlo simulations.^[^ ^94^ ^]^ Due to the dependence of self‐diffusivity on molecular residence times, the self‐diffusion was proved to be dependent on surface roughness, but the Fickian diffusion was not.

#### 3.1.4 Diffusion with Adsorption Impacts

**Figure 19:**
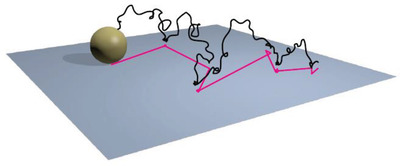
Schematic diagram of molecular surface diffusion that combines periods of immobilization with episodes of bulk diffusion above the surface. The black curve is the true 3D molecular trajectory, and the magenta line is the effective 2D trajectory across the surface. Reproduced with permission.^[^ ^95^ ^]^ Copyright 2013, American Physical Society.

Besides the confined diffusion with external physical scales discussed above, diffusion has something to do with adsorption impacts under many circumstances. For example, surface diffusion is distinguished from bulk molecular diffusion by whether gas molecules are adsorbed onto the adsorbent surface (**Figure** **19**). In this case, the dimension perpendicular to the adsorbed crystal facet would become inaccessible.^[^ ^83^ ^]^ Moreover, in porous materials with pore sizes lower than 1 nm, the surface diffusion of gas molecules plays a dominant role, leaving a low pore diffusion flux. Compared with other diffusion types, surface diffusion is thought to be driven by a chemical potential gradient on the adsorbent surface, while the diffusion barrier is related to gas adsorption energy.

The hopping (jumping) model is one of the commonly applied models for surface diffusion, in which the adsorbed molecules hop among adjacent adsorption sites on the adsorbent surfaces.^[^ ^96^ ^]^ Specifically, the mechanism of proton mobility in protic solvents could be explained by the Grotthuss proton hopping model.^[^ ^55^ ^]^ The protons are continuously adsorbed onto the solvent molecules and hop onto the adjacent sites, therefore, the protonic clusters (H_3_O^+^, H_9_O_4_^+^, H_5_O_2_^+^, etc.) are constantly created and then dissociated. Agmon elucidated that the proton mobility was an incoherent proton hopping process, and the rate‐limiting step was a cleavage of hydrogen bonds instead of proton motion or cluster growth.^[^ ^55^ ^]^ Besides proton hopping, researchers have modeled and normalized the hopping process of different particles on different interfaces. For example, the spillover of oxygen played a key role in carbon‐based catalysis, and Radovic et al. applied DFT calculation to investigate oxygen hopping on graphene surfaces, mainly focusing on prototypical clusters and periodic structures.^[^ ^97^ ^]^ A decreased hopping barrier was achieved when the graphene contained free edge sites and oxygen functioned as electronic density increased. The oxygen mobility under the most favorable circumstance was identical to that of gaseous oxygen in micropores, consistent with the commonly accepted subjective role (rather than a spectator) that the basal oxygen played in various adsorption and reaction processes of sp^2^‐hybridized carbon materials. Apart from the solid–gas interfaces, Skaug et al. further applied single‐molecule tracking at solid–liquid interfaces and found that a series of different molecules underwent intermittent random walk with non‐Gaussian displacements, contrasting the normal assumptions based on the random walk and Gaussian statistics for molecular surface diffusion.^[^ ^95^ ^]^ Moreover, the intermittent hopping was estimated to be universal for molecular surface diffusion at solid–liquid interfaces. Generally, the gas adsorption energy far exceeds the gas collision energy for most gas–solid systems, and thus the gas adsorption energy would decrease with increasing gas pressure, resulting in the enhancement of molecular surface diffusion. In addition, if gas adsorption energy and gas collision energy became comparable, an opposite trend would emerge between surface diffusivity and gas pressure. In this case, the 2D gas behavior would occur, which was manipulated by a collision among adsorbed molecules and characterized by surface $\bar{\lambda }$. Since the surface $\bar{\lambda }$ far exceeds the space distance of adjacent adsorption sites, the hopping model would be no longer applicable.^[^ ^83^ ^]^

**Figure 20:**
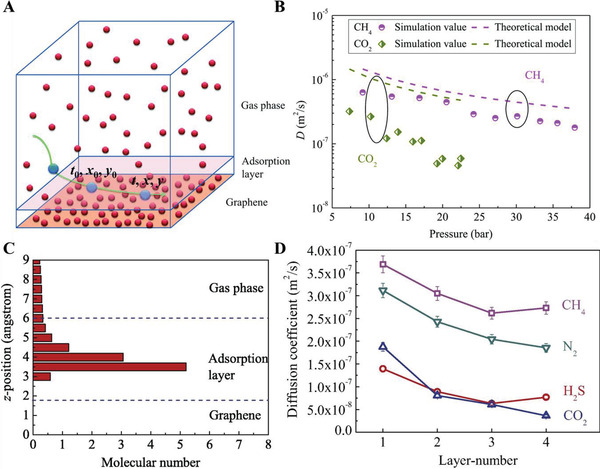
A) Model for the calculation of diffusion coefficient using the Einstein equation. B) Surface diffusion coefficients of CH_4_ and CO_2_ molecules at different pressures. The dash lines denote the theoretical values of bulk diffusion coefficients predicted using the hard sphere model based on the ideal gas kinetic theory. Reproduced with permission.^[^ ^83^ ^]^ Copyright 2017, Royal Society of Chemistry. C) Molecular number density distribution along *z*‐direction for CH_4_ adsorbed on the surface of monolayer graphene. D) Variations of diffusion coefficient versus number of graphene layers for CH_4_, H_2_S, CO_2_, and N_2_, respectively. The error bars of diffusion coefficients are plotted based on an uncertainty of 5%. Reproduced with permission.^[^ ^81^ ^]^ Copyright 2017, Elsevier.

Sun et al. investigated the surface diffusion mechanisms of different gas molecules on graphene.^[^ ^83^ ^]^ The single‐layered graphene provided weak interactions with physically adsorbed gas molecules, leaving the conventional hopping model doubtful. The surface diffusion coefficients on graphene at different pressures (**Figure** **20**A) were thus simulated and calculated by modeling CH_4_ and CO_2_ molecules with MD. According to the Einstein equation (Equation (26)), the surface diffusion coefficients were calculated as the slope of the linear relationship between mean‐square‐displacement (MSD) and time,^[^ ^83^ ^]^ in which *x* and *y* stand for the molecular coordinates at time *t*, and *x*_0_ and *y*_0_ stand for the molecular coordinates at initial adsorption time *t*_0_, respectively. The surface diffusion coefficients of both CH_4_ and CO_2_ molecules decreased as gas pressure rose (discrete dots in Figure 20B), suggesting a 2D gas behavior on the graphene surface, which was majorly manipulated by the collision of adsorbed molecules rather than the hopping model. The surface diffusion coefficients were further compared with relative bulk molecular diffusion coefficients (dash lines in Figure 20B) predicted by the hard‐sphere model (calculated according to Equation (22)). They proved qualitative correlations with each other and decreased as gas pressure rose. However, due to the confinement of other gas molecules and the interaction between gas molecules and the graphene layer, the surface diffusion coefficient was lower than the bulk molecular diffusion coefficient, especially for CO_2_ molecules with larger adsorption levels. Further investigations were carried out on multilayer graphene.^[^ ^81^ ^]^ Owing to the strong interaction between gas molecules and graphene layers, a one‐atom‐thick region of high gas density is formed above the graphene surface (denoted as the adsorption layer, Figure 20C), indicating the single layer of gas adsorption on the graphene surface. With the increase of graphene layers, the interaction between graphene and gas molecules rose, and thus the adsorption levels were enhanced. However, the adsorption levels would reach saturation when layer number was above 2, mainly because the number of effective carbon atoms capable of practically interacting with gas molecules in graphene was kept almost unchanged. As the graphene layers increased, the gas adsorption energy remained comparable with the gas collision energy, maintaining 2D gas behavior. On the other hand, the gas adsorption energy rose to increase the confinement of more gas molecules, and thus the diffusion coefficient would decrease (Figure 20D). Moreover, when the number of graphene layers was large, due to the limited interaction distance, the diffusion coefficient would be no longer significantly influenced by graphene layer numbers. The diffusivity of different gas molecules on the same graphene layer number (Figure 20D) could be further explained by the hard‐sphere models. The molecular weights and kinetic diameters of CH_4_ and N_2_ molecules were small to boost their diffusivity, while those of H_2_S and CO_2_ were larger, resulting in diffusion confinements. In addition, the adsorption levels of H_2_S and CO_2_ molecules on graphene surfaces were higher, indicating fewer gas molecules and thus a lower pressure.

(26) $$D=\frac{\langle {\left(x-x_{0}\right)}^{2}+{\left(y-y_{0}\right)}^{2}\rangle }{4(t-t_{0})}=\frac{\langle {\left(x-x_{0}\right)}^{2}+{\left(y-y_{0}\right)}^{2}\rangle }{4\Delta t}$$

#### 3.1.5 Interfacial Mass Transfer and DR‐Coupled Theory

Since a typical transport process is driven by a concentration gradient, diffusion plays a vital role in mass transfer and directing the systems that are not currently balanced into novel ones. In multi‐phase systems, the diffusion of a certain ingredient at the interface enables its mass transfer into the other phase, which is of great significance in enhancing the hetero‐contact in the two phases. One of the most acknowledged interfacial mass transfers is the Kirkendall effect (named after American chemist and metallurgist Ernest Oliver Kirkendall), illustrating the motion of metal–metal interfaces due to the mass transfer of the two metals with different diffusivity.^[^ ^98^ ^]^ The diffusion mechanisms for metal atoms were once thought to be merely position exchange among the diffused atoms with other atoms or vacancies, with no interfacial motion taking place during diffusion. However, the Kirkendall effect indicated that different metals possessed different diffusivity. Moreover, the metal–metal interface would move towards the phase with higher diffusivity and result in pores (Kirkendall porosity) during diffusion.^[^ ^99^ ^]^

**Figure 21:**
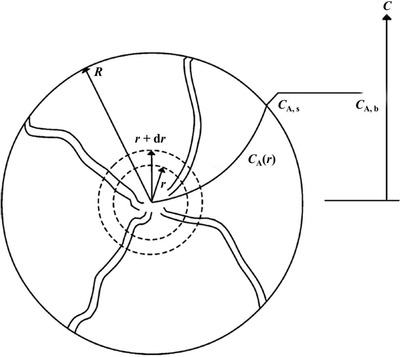
Schematic drawing of a spherical porous solid phase particle with radius *R* indicating concentration of A decreases going from the bulk (*C*_A,b_) to the surface (*C*_A,s_) and then through the pores (*C*_A_(*r*)). Reproduced with permission.^[^ ^101^ ^]^ Copyright 2005, Springer.

A more widely acknowledged form of interfacial mass transfer is that happening in multi‐phase systems, in which one or more fluid phases are contained, that is, gas–solid, or gas–liquid interfaces. This is a common circumstance for heterogeneous catalysis, gas sensing, etc. Take a gas–solid interface as an example. **Figure** **21** shows the mass transfer process of a porous solid phase material, which is based on a stagnant film model. In a typical hypothesis, the pressure gradient of the gas enables it to flow around solid particles, and a boundary layer is supposed to form between the bulk gas and the external surface of the solid phase. The fluid in this layer flows much more quiescent than that in the bulk phase, leading to a concentration gradient across the boundary layer. Gas molecules accordingly obey the diffusion instead of bulk flow routes along the boundary layer to the gas–solid interface, and then they are adsorbed onto the solid surface to undergo further processes. This process enables the transfer and accumulation of fluid molecules from the bulk fluid phase onto the solid surface, which is denoted as the “external diffusion” process.^[^ ^100^ ^]^ Moreover, when the gas molecules contact the surface of the solid phase, they may undergo several parallel processes referred to as the DR‐coupled process. During this process, some gas molecules might diffuse deeper into the solid phase to contact interior solid particles, especially for the confined diffusion along the pores in porous materials, which is denoted as the “internal diffusion” process.^[^ ^100^ ^]^ Besides, other gas molecules react inside the solid phase or just on the surface and then they are depleted. Under most circumstances, the whole process that gas molecules undergo from the bulk gaseous phase is illustrated in Figure 21, which is a combination of both external and internal diffusion driven by concentration gradients.

Due to the simultaneous occurrence between diffusion and reaction in practical DR‐coupled situations, diffusion is often denoted as its comparison with eventual reactions. Some parameters such as Mears’ criterion (*C*_m_) and Weisz–Prater criterion (*C*_WP_) were proposed to investigate the role of external and internal diffusion in the whole DR‐coupled process at gas–solid interfaces, respectively.^[^ ^100^, ^101^, ^102^ ^]^ On the other hand, the internal diffusivity of gas molecules inside an actual solid phase is smaller than that with uniformly distributed pores in optimal situations. Thus, the effective diffusivity of gas A (*D*_A,e_) inside a solid phase is defined in Equation (27), in which *ε*, *D*_A_, *σ*, and *τ* stand for the porosity of the solid phase, the diffusivity of gas A in the solid phase, construction factor, and tortuosity, respectively.^[^ ^102b^ ^]^ In practical situations, the particles in the solid phase are simplified as spheres by adopting a pseudo‐continuum pore model, and the gas molecules diffuse from the bulk gaseous phase onto the surface of the spheres and then continue to diffuse deeper inside of the pores.^[^ ^103^ ^]^

(27) $$D_{A,e}=\frac{\varepsilon D_{A}\sigma }{\tau }$$

**Figure 22:**
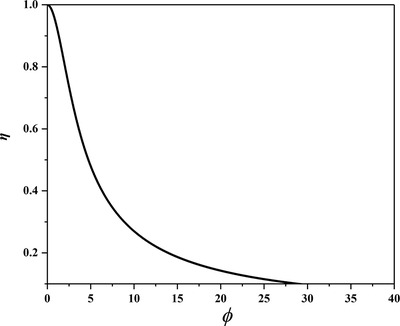
Variation of *η* with Thiele module for a spherical‐shaped catalyst following first‐order kinetics. Reproduced with permission.^[^ ^102b^ ^]^ Copyright 2007, John Wiley & Sons.

On the other hand, for porous materials with merely internal diffusion, which are equipped with a negligibly thin boundary layer, the correlation between the concentration of A and *r* can be expressed with an assumption of first‐order reaction in Equation (28), in which the dimensionless parameter *ϕ* is the ratio of reaction rate to internal diffusion rate. The parameter is also denoted as the Thiele modulus (named after American researcher E. W. Thiele), and in first‐order reactions (*n* = 1), it is calculated in Equation (29), in which *ρ*_S_ stands for apparent density.^[^ ^100^, ^102^, ^104^ ^]^ A higher Thiele modulus indicates a much faster reaction rate over the internal diffusion, and thus most A molecules react and deplete within a shallow region near the solid surface, leading to the unusable of much space inside the solid spheres. Furthermore, a lower Thiele modulus indicates a much faster internal diffusion rate over the reaction, and molecules can diffuse and react almost anywhere inside the solid spheres, and then the utility of the solid phase is enhanced.^[^ ^102b^ ^]^ Therefore, another criterion of internal effectiveness factor (*η*) is further introduced to measure the importance of internal mass diffusion resistance, which is defined as Equation (30) in the first‐order reactions inside solid spheres.^[^ ^102^, ^105^ ^]^ The internal effectiveness factor has a negative correlation with the Thiele modulus under different reaction circumstances (**Figure** **22**), which fairly provides a vivid illustration of the sphere utility. The internal diffusion inside the solid phase spheres has no resistance and the DR‐coupled process is thus surface‐reaction limited when *η* is close to 1. However, the process is internal diffusion limited when *η* is close to 0.^[^ ^102b^ ^]^

(28) $$C_{A\;}\left(r\right)=C_{A,s}\frac{\sinh\left(\phi \frac{r}{R}\right)}{\frac{r}{R}\sinh\phi }$$

(29) $$\phi =R\sqrt{\frac{k\rho_{s}}{D_{A,e}}}$$

(30) $$\eta =\frac{3}{\phi }\left(\coth\;\phi -\frac{1}{\phi }\right)$$

**Figure 23:**
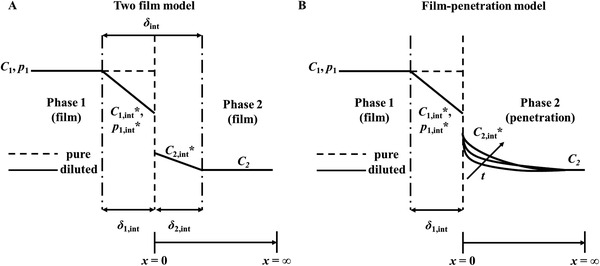
Schematic representation of concentration profiles in gas–liquid and liquid–liquid systems assuming two‐film model and combined film‐penetration model: A) Two‐film model, B) Film‐penetration model. Reproduced with permission.^[^ ^106^ ^]^ Copyright 2011, Elsevier.

Another indicative circumstance for mass transfer is that at the gas–liquid interfaces. Compared with the gas–solid interfaces, the two ingredients composing gas–liquid interfaces are both fluids, and the mass transfer of gas molecules after external diffusion and adsorption onto the liquid surface undergoes a dynamic process in a novel fluid system. In this case, several models, such as the stagnant film model and the penetration model, have illustrated the mass transfer at gas–liquid interfaces.^[^ ^106^ ^]^ The stagnant film model at the gas–liquid interfaces is similar to that in gas–solid interfaces. The only difference is that the boundary layers of the gas–liquid interfaces are present not only on the gas side but also on the liquid side. In this case, the mass transfer of gas molecules across the interface to the liquid phase obeys a novel diffusion route as it is located within the boundary layer in the gaseous phase, and a two‐film model is obtained, whose thicknesses are expressed with hydrodynamic parameters *δ*_1,int_ and *δ*_2,int_ (**Figure** **23**A).^[^ ^107^ ^]^ Although the two‐film model is simple, the prediction of hydrodynamic parameters depends on geometry, liquid agitation, and physical properties.^[^ ^106^ ^]^ On the other hand, the penetration model assumes that the liquid phase is not stationary, and the flux of gas molecules into the liquid flux is no longer steady. Instead, the liquid in contact with the gaseous phase changes after a period (exposure time), during which it is replaced by fresh liquid from the bulk phase.^[^ ^108^ ^]^ The exposure time (*θ*) is measured by hydrodynamic properties (e.g., the interface velocity and its length).^[^ ^106^ ^]^ Since the surface of the gas–liquid interface changes constantly, the penetration model is denoted as the surface renewal model as well.^[^ ^109^ ^]^ Toor et al. further derived a novel film‐penetration model based on the combination of the stagnant film model and the penetration model (Figure 23B).^[^ ^110^ ^]^ The penetration model for dynamic systems seems more realistic than the stagnant film model, and in practical situations, the above models are usually combined with the fluid flow models in determining the performance of the overall mass transfer,^[^ ^106^ ^]^ resulting in several modified modeling investigations.^[^ ^111^ ^]^

Similar to Equation (28), when the first‐order reaction is assumed, the concentration of gas molecule A in the liquid phase at a specific depth *x* from the interface can be expressed in Equation (31), in which *C*_A,i_, and *L* stand for the concentration of A at the gas–liquid interface (similar to *C*_A,s_ at gas–solid interfaces) and the whole thickness of the film in the liquid phase, respectively. Hatta number is another dimensionless parameter, which is expressed in Equation (32) and introduced to simplify the equation.^[^ ^106^, ^107^ ^]^ Similar to Thiele modulus, the Hatta number compares the reaction rate in the film with the diffusion rate through the film.^[^ ^112^ ^]^ If *Ha* > 3, the reaction rate is fast and a vast majority of gas molecules react within the liquid film near the interface, resulting in a diffusion‐limited process. Whereas if *Ha* < 0.3, the diffusion rate is high enough for gas molecules to react in the bulk liquid phase, leaving a reaction‐limited process.^[^ ^106^ ^]^ A liquid utilization factor (*η*_L_) is applied to further investigate the impacts of diffusion and determine the utility of the liquid film at gas–liquid interfaces. It is defined as the ratio of the actual reaction rate to the optimal one in the absence of concentration gradients, which is expressed in Equation (33), in which *k*_c_ stands for mass transfer coefficient.

(31) $$C_{A}\left(x\right)=\frac{C_{A,i}\sin h\left[\mathrm{Ha}\left(1-\frac{x}{L}\right)\right]+C_{A,b}\sin h\left(\frac{x}{L}\right)}{\sin h\mathrm{Ha}}$$

(32) $$Ha=x\sqrt{\frac{k}{D_{A,e}}}$$

(33) $$\eta_{L}=\frac{D_{A,e}}{\mathrm{Ha}k_{c}L\tan h\mathrm{Ha}}\left(1-\frac{C_{A,b}}{C_{A,i}\cos h\mathrm{Ha}}\right)$$

### 3.2 Diffusion Models for MOS Gas Sensors

The process of gas diffusion can act as a supplement to common adsorption/desorption theories, which are mainly based on the physical/chemical properties of MOS materials. Although the gas diffusion mechanisms primarily play an auxiliary role, the manipulation of gas diffusion has a great impact on gas responses. In addition, the external diffusion of gas molecules takes place before adsorption onto MOS materials, which is usually neglected due to its few contributions to sensor responses. After the adsorption of gas molecules on MOS gas sensors, many gas molecules undergo internal diffusion inside the MOS materials and then a DR‐coupled process, during which some of them are immobilized due to adsorption or reaction. The basic assumption is that the rate of immobilization is higher than that of gas diffusion, and thus two different types of immobilized molecules and free molecules are produced. The chemical reaction immobilizes some gas molecules to slow down their diffusion rate. Based on Fick's second law (Equation (19)), Crank gave the modified diffusion equation (Equation (34)), in which *C*_A_ and *S*_A_ stand for the concentrations of free and immobilized A molecules, respectively.^[^ ^113^ ^]^

(34) $$\frac{\partial C_{A}}{\partial t}=\nabla \left(D\nabla C\right)-\frac{\partial S_{A}}{\partial t}$$

#### 3.2.1 Gardner's Linear and Non‐Linear DR Models

**Figure 24:**
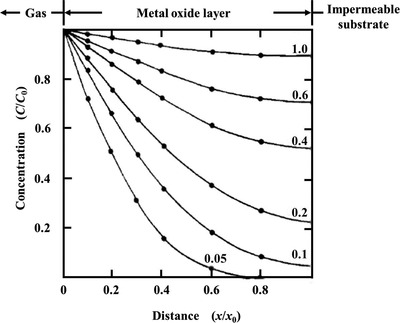
Analytical (full curve) and numerical (dot) solution of the DR equation showing several concentration profiles in a homogeneous oxidic layer at various instants in time (expressed as a fraction of diffusive time constant). Reproduced with permission.^[^ ^9^ ^]^ Copyright 1989, IOP Publishing Ltd.

In 1989, Gardner developed a linear DR model,^[^ ^9^ ^]^ in which *S*_A_ is estimated to be proportional to *C*_A_. Thus, Equation (34) can be simplified into Equation (35). The correlation between gas concentration and diffusion depth (**Figure** **24**) shows that the curves followed a sharp decrease as distance increases in low *T* values, and followed a steady trend in high *T* values, indicating the reduction of concentration gradient as well as diffusion. However, this model assumed that the diffusion rate is lower than the reaction rate, so it was only feasible in thick and porous layers, confining its application scope.

(35) $$\frac{\partial C_{A}}{\partial t}=D_{A,e}\nabla^{2}C_{A}$$

In 1990, Gardner developed a non‐linear DR model.^[^ ^62^ ^]^ Compared with the former one, in this model, *S*_A_ is assumed to be proportional to *C*_A_ to the power of *r* (Equation (36)), in which *B* and *r* stand for the constants depending on sensing materials and detected gas, respectively. In this case, the diffusion equation can be simplified as Equation (37). The assumption of non‐linear correlation extended the application range of this model, and in 1996 Vilanova et al. derived the conductance transients based on this model.^[^ ^10b^ ^]^ The research of conventional selectivity was based on different gas sensing responses towards different gas molecules, but the method based on the response time was introduced in their research. The equilibrium conductance changes Δ*G*(∞) for 20 ppm of benzene and 50 ppm of *o*‐xylene were similar, but their normalized response time values *τ*’ were different, mainly due to different diffusion abilities. Thus, the two kinds of gas molecules could be well distinguished. Furthermore, Lu et al. proposed that the DR‐coupled process was also included in gas sensing experiments, which steadily established the application of the gas diffusion model in gas sensing research.^[^ ^114^ ^]^

(36) $$S_{A}=BC_{A}^{r}$$

(37) $$\frac{\partial S_{A}}{\partial t}=\frac{D_{A}}{B^{\frac{1}{r}}}\;\frac{\partial^{2}S_{A}^{\frac{1}{r}}}{\partial x^{2}}$$

#### 3.2.2 Yamazoe's Diffusion Models

In 2001, Yamazoe et al. systematically introduced the DR‐coupled theory and gas diffusion‐controlled sensing sensitivity into the gas sensing of SnO_2_.^[^ ^10c^ ^]^ The model is based on two major assumptions:

a) Knudsen diffusion would happen in SnO_2_ films;
b) diffusion would obey the first‐order kinetics.

**Figure 25:**
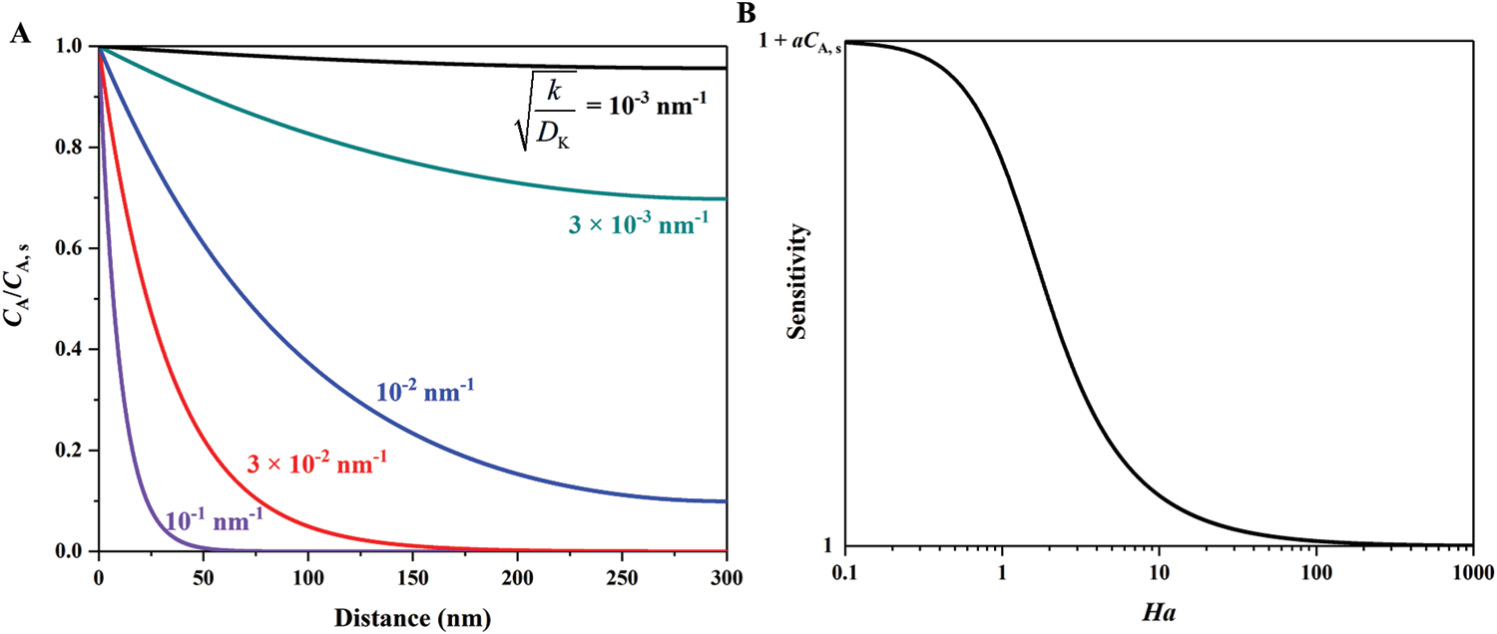
A) Simulated gas concentration profiles inside a sensing film (thickness 300 nm) for various values of $\sqrt{\frac{k}{D_{K}}}$ at fixed temperature; B) generalized expression of the gas sensitivity of thin films at a fixed temperature in terms of non‐dimensional parameter, Hatta number *Ha*. Reproduced with permission.^[^ ^10c^ ^]^ Copyright 2001, Elsevier.

In the first assumption, the SnO_2_ grains with a pore size of about 10–15 nm were synthesized from a sol suspension by spin coating, and the pore size was in the range for Knudsen diffusion. Based on the two assumptions and the DR‐coupled models, the Knudsen diffusion equation is expressed as Equation (38), in which *C*_A_, *x*, and *k* stand for the concentration of target gas, diffusion depth, and rate constant of reaction, respectively. Equation (38) equals zero at the steady state. By introducing boundary situations and the Hatta number (*Ha*, Equation (32)), the particular solution is expressed as Equation (39), in which *C*_A,s_, and *L* stand for the surface concentration and the SnO_2_ layer thickness, respectively.^[^ ^32^ ^]^ As mentioned above, *Ha* illustrates the competitive relationship between diffusion and reaction in DR‐coupled processes (**Figure** **25**A). Larger *Ha* values indicate higher activity in reaction with reduced diffusion depth, and smaller values boost diffusion behaviors. What's more, another assumption is made that the conductance change of the SnO_2_ sensing layer is linear to the gas concentration under exposure to the gas (Equation (40)), in which *σ*(*x*) and *σ*_0_ stand for conductance under exposure to the gas and in air, respectively, and *a* is a constant. The assumption of linearity is made under the circumstances of low gas concentrations, in which the validity may be challenged under other circumstances. By integrating conductance, the response (gas sensitivity) of the film (*S*) is defined as Equation (41). Specifically, the Hatta number contains three main factors in the field of gas sensing including reaction rate, Knudsen diffusion coefficient, and layer thickness. An extremely low *Ha* (close to zero) indicates a much faster internal diffusion rate compared with the reaction rate, resulting in uniform gas distribution in the MOS sensing layer with almost no concentration gradient and little impact on gas diffusion throughout the whole system. When the *Ha* values increase, the internal diffusion rate gradually becomes comparable with the reaction rate, and then plays a more dominant role. Practically, in the DR‐coupled process of MOS gas sensing, comparable internal diffusion and reaction rates would make the best use of the gas sensor, in which the changing tendency of the sensor's response or sensitivity becomes most conspicuous. As shown in Figure 25B, this phenomenon happens in the *Ha* range of 1–10. The *k* and *D*_K_ values remained constant for a specific gas at a certain temperature, and thus the thickness of the sensing layer *L* could be precisely manipulated to meet the *Ha* value, suggesting a feasible way of quantitative experiment design. Both two main parameters, Knudsen diffusion coefficient, and reaction rate correlate with temperature, and the rise of temperature can boost both diffusion and reaction rates. Although both *D*_K_ and *k* have a positive correlation with temperature, the latter increases much faster than the former. Thus, the reaction is dominant at higher temperatures, and diffusion is the rate‐limiting step, while at lower temperatures vice versa.

(38) $$\frac{\partial C_{A}}{\partial t}=D_{K}\frac{\partial^{2}C_{A}}{\partial x^{2}}-kC_{A}$$

(39) $$C_{A}=C_{A,s}\frac{\cos h\left[\left(L-x\right)\sqrt{\frac{k}{D_{K}}}\right]}{\cos h\left[L\sqrt{\frac{k}{D_{K}}}\right]}=C_{A,s}\frac{\cos h\left(1-\frac{x}{L}\right)\mathrm{Ha}}{\cos h\mathrm{Ha}}$$

(40) $$\sigma \left(x\right)=\sigma_{0}\left(1+aC_{A}\right)$$

(41) $$\begin{array}{c} S=\frac{R_{a}}{R_{g}}=1+\frac{aC_{A,s}}{L\sqrt{\frac{k}{D_{K}}}}\tan h\left(L\sqrt{\frac{k}{D_{K}}}\right)=1+\frac{aC_{A,s}}{\mathrm{Ha}}\tan h\mathrm{Ha} \end{array}$$

Although this model is relatively formalized, the limitations still confine its applications. Most of the limitations originate from two assumptions: Knudsen diffusion in SnO_2_ layers and conductance correlations (Equation (40)). However, the existence of surface diffusion in mesoporous materials is neglected in Knudsen diffusion. Research on ordered mesoporous silica fibers revealed that at room temperature surface diffusion only accounts for about 10% of whole diffusive flux, indicating the enhancement of Knudsen diffusion in ordered mesoporous materials.^[^ ^115^ ^]^ On the other hand, the main shortage of this model is that the assumption was just elucidated without any supportive experimental data. Although Yamazoe et al. elucidated the theoretical basis for the power law of MOS sensors mentioned above,^[^ ^10^, ^66^ ^]^ the diffusion model was not modified to fit the law.^[^ ^116^ ^]^ Another limitation resulted from the first‐order kinetics assumption of gas diffusion, which proved to be invalid in doped materials (e.g., PdO) or in the presence of humidity.^[^ ^117^ ^]^

**Figure 26:**
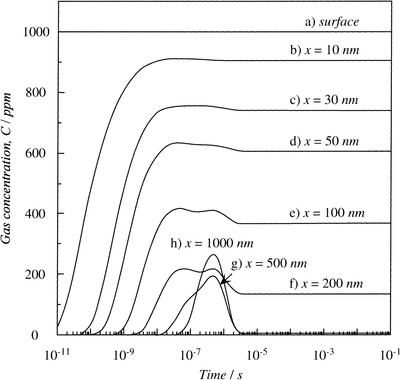
Time courses of gas concentration at various depths (*x*) in the sensing film. Reproduced with permission.^[^ ^10d^ ^]^ Copyright 2002, Elsevier.

In 2002, Yamazoe et al. further extended the diffusion model to the non‐steady state (Equation (42)).^[^ ^10d^ ^]^ Compared with Equation (38), gas concentration is also related to diffusion time in the non‐steady state. A typical factor in a non‐steady state is the overshooting phenomenon (**Figure** **26**), indicating the competition in DR‐coupled processes. Since the internal diffusion of gas molecules occurs earlier than reactions in the close vicinity of the surface, the gas concentration is overshot to a higher value than that at the equilibrium, and then steadily drops to the equilibrium due to reaction consumption. The origination of overshooting phenomenon is the non‐equilibrium state of local concentration resulting from the different rates between internal diffusion and reaction of gas molecules. In Matsunaga's experiments,^[^ ^10d^ ^]^ the time scale of overshooting phenomenon is ≈10^−6^ s, which is undetectable by the naked eye or instruments. However, in practical experiments, especially under some extreme conditions of *D*_A_ and/or *L*, a small but detectable peak may occasionally be found before entering equilibrium. Then a non‐level response–recovery curve was obtained,^[^ ^28^ ^]^ which might be eliminated by adjusting experimental operations or parameters. In practical sensing experiments, the gas molecules undergo external diffusion in the air before adsorption and internal diffusion inside the MOS sensing layer. Although the external diffusion has few contributions to sensor responses and is thus usually negligible, the boosted rate of adsorption also reversely contributes to the desorption rate on injecting large amounts of test gas. The gas molecules are released into the air again and then go on to another external diffusion process. In this case, the impact of external diffusion must be considered, and the system undergoes a tripartite equilibrium consisting of rapid surface adsorption/desorption, internal diffusion, and reaction. During this process the local non‐equilibrium gas concentration changes swiftly, resulting in a slight or even sharp change in resistance and one or even more overshooting peaks before eventual equilibrium.

(42) $$\frac{\partial C_{A}\left(x,t\right)}{\partial t}=D_{A}\frac{\partial^{2}C_{A}\left(x,t\right)}{\partial x^{2}}-kC_{A}\left(x,t\right)$$

In 2003, Yamazoe et al. studied gas diffusion dynamics in response and recovery and developed a new diffusion model.^[^ ^10e^ ^]^ Compared with the former models, this model does not contain any assumptions in the derivation, which is claimed to be valid throughout the whole DR process. According to the diffusion equation (Equation (42)), the gas concentration can be split into the sum of a homogeneous function and another inhomogeneous one. Although this model is not based on any assumptions that confine its application range, it merely accounts for diffusion constant and reaction rate and neglects the impacts of temperature and layer thickness, which play an important role in transient behavior.^[^ ^118^ ^]^ The experimental studies elucidating the reduction of response time in bimodal meso–macroporous structures have confirmed this point of view.^[^ ^119^ ^]^

**Figure 27:**
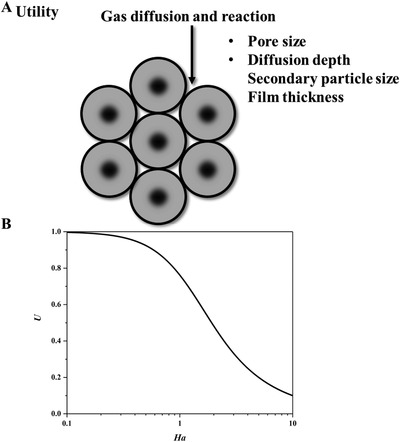
A) Utility factor as well as the physicochemical or materials properties involved for MOS gas sensors; B) utility factor as correlated with *Ha*. Reproduced with permission.^[^ ^121^ ^]^ Copyright 2005, Elsevier.

Three basic factors, including receptor function, transducer function, and utility factor, are thought to affect gas sensing properties.^[^ ^65^, ^120^ ^]^ In 2003, Yamazoe introduced the utility factor (originated from the liquid utilization factor in Equation (33)) into MOS gas sensing (**Figure** **27**A) to evaluate the DR‐coupled effect in sensing layers, which concerned the accessibility of target gas to inner oxide grains.^[^ ^32^, ^121^ ^]^ The volcano‐type curve between sensor responses and the working temperature had been observed before, but the work on SnO_2_ thin films with quantitative results was not achieved.^[^ ^10^, ^121^ ^]^ Specifically, the utility factor of a sensor (*U*, Equation (43)) is defined as the ratio of actual response (*S*) to the optimal intrinsic response (*S*_i_) at *Ha* = 0. Figure 27B shows the correlation between *U* and *Ha*, showing a similar reverse‐S curve to that of Figure 25B, in which both the independent variables are *Ha*. The *Ha* value should be kept low to increase the utility factor, indicating that lower layer depth *L* and *k*/*D*_K_ ratio make sense, which has been proved by previous experiments.^[^ ^10c^ ^]^ Moreover, since *D*_K_ is proportional to pore radius *r* (Equation (25)), in which the latter has a roughly positive correlation with the size (*D*) of grains, the utility factor can be also manipulated by adjusting the grain sizes.

(43) $$U=\frac{S}{S_{i}}=\frac{\tan h\mathrm{Ha}}{\mathrm{Ha}}$$

#### 3.2.3 Further Theoretical Diffusion Models and Modifications

**Figure 28:**
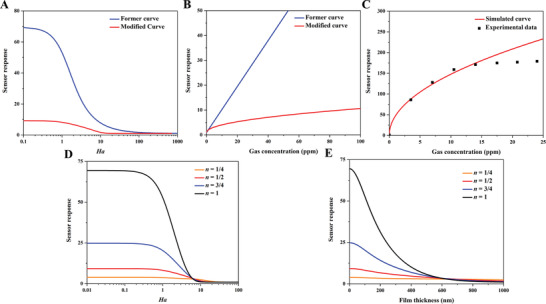
A) Sensor response as a function of *Ha* simulated by the former expression and modified expression; B) Sensor response as a function of gas concentration simulated by the former expression and modified expression; C) Correlation of the modified expression with experimental sensor response of thin films in different gas concentration; D) Sensor response in reducing gas as a function of *Ha* at various power law exponent (*n*) values; E) Relationship between sensor response and film thickness at various values of power law exponent (*n*) in reducing gas at a fixed temperature. Reproduced with permission.^[^ ^61b^ ^]^ Copyright 2009, Elsevier.

The above diffusion models have been widely applied in gas sensing experiments, but some shortages or limitations still exist, and thus researchers have been trying to improve them. In 2009, Liu et al. proposed a modification for the diffusion model.^[^ ^61b^ ^]^ Based on H_2_S sensing experiments in SnO_2_ thin films, it was found that the linear relationship of conductance and gas concentration (Equation (40)) did not satisfactorily fit the experimental results, and a power law exponent of 0.5 was found instead. Compared with the former model, the curves of sensor response versus *Ha* or gas concentration in the new model become gentler (**Figure** **28**A, B), mainly due to a decrease in the power law exponent. In the original model mentioned above, the changing tendency of gas response becomes most conspicuous in the *Ha* range of 1–10, which may be feasible in the design of a quantitative experiment. However, the tendency is weakened in the new model, indicating less dependence on the sensor response with the gas concentration. Although the simulated curve originated from the new model fits some experimental data, the experimental plots are still derived in H_2_S concentrations higher than 10 ppm (Figure 28C). In this case, the response enters saturation, and the conductance is high enough to affect the power law exponent. The power law exponent was tested to have a change due to target gas and sufficient high concentration.^[^ ^61a^ ^]^ As a result, the new model was extended to an even broader range, in which the parameter of power law exponent *n* was considered. In this way, the conductance and the gas response are expressed in Equations (44) and (45). Similarly, for *n* values from 0.25 to 1 (when *n* = 1, it belongs to the original diffusion model), the changing tendency of sensor response with parameters like *Ha* or film thicknesses (Figure 28D,E) becomes more conspicuous, leading to the significant increase of gas diffusion in the whole gas sensing system.

(44) $$\sigma \left(x\right)=\sigma_{0}\left\{1+a{\left[C(x)\right]}^{n}\right\}$$

(45) $$S=1+\frac{a(6+\mathrm{nH}a^{2})}{6}{\left(\frac{C_{A,s}}{\cos h\mathrm{Ha}}\right)}^{n}$$

**Figure 29:**
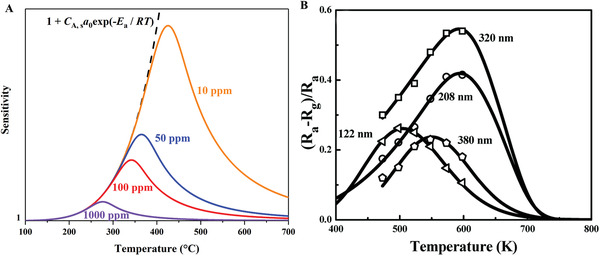
A) The dependence of gas sensitivity on temperature at various film thicknesses, simulated under the conditions: *E*_a_ = 50 kJ mol^−1^, *E*_k_ = 200 kJ mol^−1^, *A* = 1.7 × 10^7^ nm^−1^ K^1/4^, and *a*_0_ = 3400 ppm^−1^. Reproduced with permission.^[^ ^10c^ ^]^ Copyright 2001, Elsevier. B) Experimentally obtained temperature variation of CO response (500 ppm) for film thicknesses ranging from 122 to 380 nm (symbols), and the solid lines are fitted non‐linearly. Reproduced with permission.^[^ ^64^ ^]^ Copyright 2017, Royal Society of Chemistry.

In 2017, Ghosh et al. elucidated a similar model including non‐linear variation between conductance and gas concentration.^[^ ^64^ ^]^ It was originally concluded that the gas response and operating temperature corresponding to the maximum response (*T*_opt_) decreased as film thickness increased (**Figure** **29**A).^[^ ^10c^ ^]^ However, by carrying out a series of CO sensing experiments, it was found that the gas response and *T*_opt_ increased with increasing the layer thickness to the maximum value (320 nm), and then dropped to lower values (Figure 29B). Moreover, the response variation with temperature for different *n* values was also simulated, which shared similar results with the report by Liu et al.^[^ ^61b^ ^]^

#### 3.2.4 Manipulation of Pore Properties in Practicing DR‐Coupled Models

Besides establishing diffusion models based on mathematical and physical equations, researchers who are experts in material design are keen on improving gas diffusion abilities in MOS gas sensors as well. As mentioned above, the confined diffusion of gas molecules inside the pores of a porous sensing layer is a typical process for internal diffusion within a gas sensor.^[^ ^93c^ ^]^ Therefore, the manipulation of pore properties (pore sizes, pore morphologies, etc.) is a logical thought for diffusion adjustment. Pore size is commonly related to diffusivity itself as well as specific surface area. Higher diffusivity in larger pores results in lower specific surface areas, while smaller pores with higher specific surface areas confine gas diffusion.^[^ ^33^ ^]^ Thus, decent pore size is a must in practicing the DR‐coupled model.

**Figure 30:**
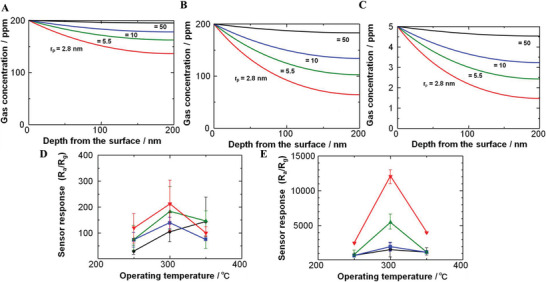
A–C) Simulated concentrations of (A) H_2_, (B) CO, and (C) H_2_S as a function of depth from the surface of films with different pore radii. D,E) Sensor responses to (D) CO and (E) H_2_S as a function of operating temperature, and closed circles, squares, triangles, and inverted triangles correspond to samples with crystallite sizes of 7, 11, 14, and 18 nm, respectively. Reproduced with permission.^[^ ^122^ ^]^ Copyright 2013, American Chemical Society.

Kida et al. synthesized a series of SnO_2_ nanoparticles with controllable grain sizes and carried out gas‐sensing research for H_2_, CO, and H_2_S.^[^ ^122^ ^]^ The pore sizes on sensing layers were manipulated by controlling grain sizes of SnO_2_ nanoparticles. Compared with CO and H_2_S molecules, the concentration of H_2_ is less dependent on the depth from the surface of the sensing layer (**Figure** **30**A), due to the highest diffusivity of H_2_ molecules with the smallest molecular weight, and the utility factor of the SnO_2_ sensing layer can also be improved. In other words, the pore sizes of sensing materials have little impact on the diffusion of H_2_ molecules. On the other hand, due to the lower diffusivity of CO and H_2_S molecules, sensing materials with larger grain sizes and pore sizes are needed to increase their diffusion rates (Figure 30B, C) and the utility factor. Therefore, materials with smaller pores may improve selectivity in H_2_ sensing experiments. Moreover, although the Knudsen diffusion coefficient values (*D*_K_) of CO and H_2_S are similar (according to Equation (25), the *D*_K_ value of CO is about 1.05 times of H_2_S), the response of H_2_S is far higher than CO, mainly resulting from the high surface reaction activity of CO (Figure 30D, E). The CO molecules with high activity may be consumed just at the surface of SnO_2_, and the diffusion to deeper areas is thus confined, leading to a decrease in the utility factor of the sensing layer. In comparison, the H_2_S molecules with lower reaction activities can increase the utility factor and sensor response by diffusing to deeper areas. Therefore, it is important to improve the utility factor by combining gas diffusion with reactions.

**Figure 31:**
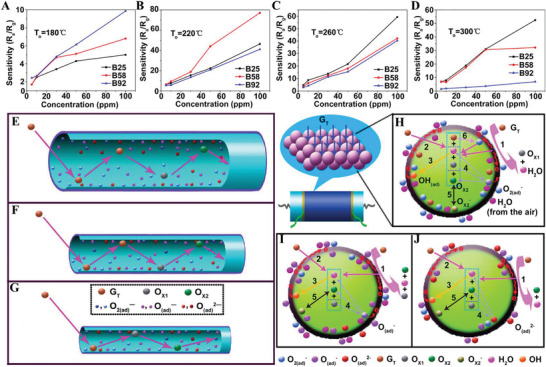
A–D) Concentration–response curve of acetone at different operating temperatures. The three materials B25, B58, and B92 are named after their specific surface areas. (A) 180, (B) 220, (C) 260, and (D) 300 °C. E–G) Schematic illustration of the target gas diffusion process in different pore sizes: (E) large, (F) medium, and (G) small. H–J) Schematic illustration of the target gas diffusion process in the SnO_2_ hollow microspheres at different temperatures. (H) < 150, (I) 150–200, and (J) > 200 °C. Reproduced with permission.^[^ ^82^ ^]^ Copyright 2015, American Chemical Society.

Wang et al. put forward a model to evaluate the relationship among all aspects of the gas sensing properties, that is, the surface chemical reaction (SCR), gas diffusion, operating temperature corresponding to the maximum response (*T*_opt_), target gas, etc. When the *T*_opt_ is lower than 300 °C, gas diffusion and SCR play the key role in elucidating the gas sensing mechanism.^[^ ^82^ ^]^ The SCR rate is low at lower temperatures (**Figure** **31**A), which thus becomes the rate‐limiting step and follows the surface‐control theory. Therefore, materials with larger specific surface areas show higher responses. The increase in diffusion rate is not as high as SCR at higher temperatures (Figure 31C, D), which follows the diffusion‐control theory, and the materials with larger pore sizes show higher responses. At intermediate temperatures both gas diffusion and SCR are equal in competing (Figure 31B), resulting in the formation of a dually controlled stage, and the materials with intermediate structure express the best responses. In a word, the SCR control is replaced by diffusion control as the temperature goes up. After that, a novel pore canal model was elucidated (Figure 31E–G) to illustrate the Knudsen diffusion process of gas molecules in materials with different pore sizes. At low temperatures, the mean free path of gas molecules is higher than pore sizes, thus confining gas molecules from diffusion into the materials and happening in exterior places for most sensing processes. Both the diffusion and SCR rates are low, but the latter dominates the process. As the temperature goes up, the mean free path gradually decreases, which is still comparable to pore sizes, therefore, more molecules can diffuse into interior sites to react with oxygen molecules inside the pores. At higher temperatures mean free path becomes smaller than pore sizes, which encourages more molecules to diffuse. However, the SCR rate increases much faster than the diffusion rate, resulting in the diffusion control process. Another hollow sphere model (Figure 31H–J) was elucidated to illustrate the relationship between SCR and gas diffusion. At lower temperatures (Figure 31H), the adsorbed oxygen on the SnO_2_ surface is mainly inactive O_2_^−^, which thus limits the SCR rates. Some gas molecules are partially oxidized on the surface, and others diffuse into the interior sites, which undergo a series of different oxidation reactions, including partial oxidation, complete oxidation, and ionization. At transition state (Figure 31I) or higher (Figure 31J), the oxygen ions are mainly present in the form of more active O^−^ and O^2−^. The SCR becomes more active, which enables full oxidation of gas molecules inside the spheres, and the gas diffusion turns into the rate‐limiting step.

**Figure 32:**
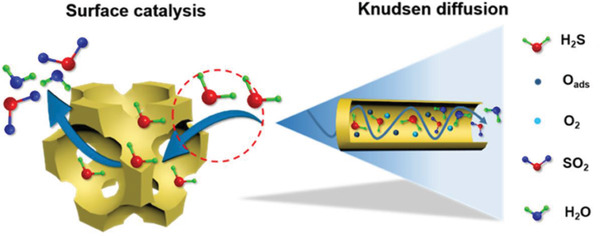
Surface catalysis and Knudsen diffusion model between H_2_S and Ce‐2/mWO_3_. Reproduced with permission.^[^ ^51f^ ^]^ Copyright 2022, American Chemical Society.

In addition, the precise manipulation of sensor morphologies can make further contributions to higher sensing abilities. In the case of MOS materials, the synthesis of ordered mesoporous metal oxide sensing materials is a promising route. The precise manipulation of pore structures (pore size, volume, crystallinity, wall thickness, and symmetry) is critical to promoting higher responses or selectivity.^[^ ^123^ ^]^ Our group has long been developing highly ordered mesoporous metal oxide sensing materials via the templating method, based on various structure‐directing agents including soft templates (amphiphilic block copolymer) poly(ethylene oxide)‐block‐polystyrene (PEO‐*b*‐PS) and polystyrene‐block‐poly(4‐vinylpyridine) (PS‐*b*‐P4VP),^[^ ^51^, ^92^, ^93^, ^124^ ^]^ as well as hard templates (urea–formaldehyde, UF).^[^ ^51c^ ^]^ Specifically, in the soft template route, based on the evaporation‐induced self‐assembly (EISA) strategy,^[^ ^125^ ^]^ the field of mesopore engineering has been established with various pore sizes and morphologies. Now that the pore sizes of the uniformly mesoporous materials are close to the mean free path of most test gases, they are favorable for the transport and diffusion of gas molecules with high specific surface areas and interconnected mesopores.^[^ ^115^, ^124^ ^]^ Liu et al. fabricated Ce‐doped mesoporous WO_3_ gas sensors (denoted as Ce‐2/mWO_3_) for H_2_S detection, in which the model of surface catalysis and Knudsen diffusion was proposed for mechanism elucidation (**Figure** **32**).^[^ ^51f^ ^]^ The H_2_S molecules undergo Knudsen diffusion through the mesopores before cooperating in surface catalysis reactions at rich active sites. The mesostructured sensing materials promoted the diffusion of H_2_S molecules into the inner structure and accelerated the formation of a depletion layer and the transportation of charge carriers. On the other hand, the surface catalysis process enabled the oxidation of H_2_S molecules into both gaseous SO_2_ and solid WS_2_. The production of WS_2_ with a narrower band gap and lower resistance than the original WO_3_ further enhanced sensor responses.

#### 3.2.5 Enhanced Gas Diffusion in Pulse‐Driven Gas Sensors

**Figure 33:**
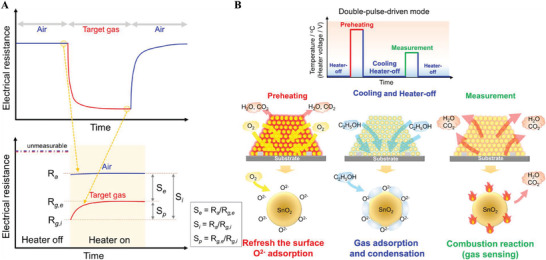
A) Determination of the sensor responses: *S*_e_, *S*_i_, and *S*_p_. Reproduced with permission.^[^ ^131b^ ^]^ Copyright 2020, American Chemical Society. B) Schematic model of gas detection based on double‐pulse‐driven mode using SnO_2_‐based semiconductor gas sensors. Reproduced with permission.^[^ ^131c^ ^]^ Copyright 2020, American Chemical Society.

Although the gas diffusion theory still needs to be optimized through quantitative analysis and modeling,^[^ ^28^ ^]^ it is of great significance in gas sensing research and has been widely applied in explaining the response mechanisms of MOS sensing materials with different morphologies.^[^ ^73^, ^126^ ^]^ In recent years, some novel approaches have been developed to improve sensing performance,^[^ ^93c^ ^]^ including optical gas sensor,^[^ ^5^, ^7^, ^127^ ^]^ surface plasmon resonance (SPR)‐enhanced gas sensor,^[^ ^128^ ^]^ pulse‐driven gas sensor,^[^ ^129^ ^]^ and FET gas sensors,^[^ ^59^, ^130^ ^]^ among which pulse‐driven micro‐gas sensors can contribute to gas diffusion improvements. Shimanoe et al. applied a pulse‐driven heating sensor in the detection of VOCs, in which gas molecules diffuse deep into the sensing layers, then condensate at the heater‐off phase, and finally undergo combustion reactions at the heater‐on phase.^[^ ^129^, ^131^ ^]^ It is worth mentioning that the popularity of pulse‐driven micro‐gas sensors among researchers is initially due to their low‐power‐consumption.^[^ ^93^, ^129^ ^]^ However, the gas diffusion abilities are also improved in pulse‐driven mode. In conventional situations, external heating is applied throughout the whole sensing process, and the gas molecules may combust near the surface of the sensing layer at high temperatures, resulting in low utility factors and responses. However, the pulse‐driven heating sensor enables the permeation of gas molecules deep into the sensing layer even at high operating temperatures, and thus the utility factor of the sensor rises.^[^ ^129b^ ^]^ Furthermore, the novel definition of responses at different stages (initial, eventual and their proportion, denoted as *S*_i_, *S*_e_, and *S*_p_, respectively, **Figure** **33**A) provided a vivid view of the different steps that the gas molecules undergo during the sensing processes (diffusion, condensation, and combustion), and illustrated clear response and selectivity of the sensor on VOC molecules against others.^[^ ^131a,b^ ^]^ The introduction of a preheating procedure before the pulse‐driven heating (denoted as double‐pulse‐driven mode, Figure 33B) further uplifted the sensing performance by a surface modification to facilitate the adsorption of O^2−^ ions, which helps to achieve the detection of ppt‐level VOCs.^[^ ^131c,131d^ ^]^

## 4 Conclusions and Perspective

In the field of MOS gas sensors, most efforts have been primarily focused on the manipulation of the chemical reaction between gas molecules and sensitive materials. However, the dynamic behavior of gas molecules, including adsorption, desorption, and diffusion, are also closely related to the sensing process. The adjustment of adsorption, desorption, and diffusion processes is beneficial to the managing by the sensing process and even sensor performance, including sensitivity, selectivity, and response/recovery dynamics, which is vital to developing high‐performance gas sensors.

Oxygen adsorption is the most common phenomenon when the sensing materials are exposed to the air, and this can lead to EDLs or HALs and cause resistance variations. Further adsorption takes place between tested gas and adsorbed oxygen, resulting in different responses. In oxygen‐free situations, chemical adsorption of reducing gas molecules and reaction with lattice oxygen can cause even higher responses. When the test gas reacts directly with the sensing material, the chemical adsorption can either boost the response or show a turnover. Both chemical adsorption and physical adsorption, and even co‐adsorption can happen in the presence of water molecules, which mainly cause a barrier for higher responses at low temperatures. Physical adsorption may be also dominant for oxygen adsorption at room temperature, causing a slight increase in resistance. Based on the combination of the surface depletion theory of the MOS materials and the change in resistance caused by adsorption, the theoretical basis for the power law between resistance and partial pressure of the test gas was eventually elucidated. Moreover, the dynamics in response/recovery processes were discussed in depth, in which adsorption/desorption processes acted as the rate‐limiting steps.

Although gas diffusion plays an auxiliary role in gas sensing, the manipulation of diffusion also helps to improve both sensing response and selectivity. Overall, the reaction of gas molecules with the MOS sensing layer follows the DR‐coupled process. With the increase in pore size of the sensing materials, gas molecules follow the surface, Knudsen, and molecular diffusion. In Knudsen diffusion, gas molecules are more likely to collide with active sites and then react with them. Among the materials with different nanostructures, the ordered mesoporous materials are prone to follow the Knudsen diffusion. Assuming Knudsen diffusion and first‐order kinetics, a series of gas diffusion models has been established and optimized, and they have proved validity in various experiments, but further quantitative analysis is still needed. As temperature rises, the gas–solid interface reaction, instead of the diffusion, becomes the dominant process, leaving the diffusion to be the rate‐limiting step. Higher temperature decreases the mean free path of gas molecules and enables the activation of both gas molecules and sensing materials, but it decreases the gas responses. Larger pore sizes of the sensing materials can enhance diffusion abilities, but their corresponding low specific surface areas are unfavorable to improving the reaction rate. For gas molecules with high diffusivity (H_2_), restricting pore sizes may be feasible in improving their selectivity. As a novel testing mode, pulse‐driven gas sensors split diffusion and reaction processes apart, promoting gas diffusion at high temperatures and improving sensor utility.

It is well known that an ideal MOS gas sensor should exhibit excellent comprehensive performance in terms of high response and selectivity, fast response/recovery time, and low cost. The dynamic processes of adsorption/desorption and diffusion play a significant role in the field of material design for better sensor fabrications. Based on this review, some perspectives can be expected to further improve gas adsorption/desorption and diffusion toward high‐performance MOS gas sensors. i) The gas diffusion models can be further improved. At present only the internal diffusion impacts during the whole sensing process have been considered. This is consistent with the assumption that gas adsorption marks the “initial” of a sensing process. However, the external diffusion process between gas injection and adsorption has impacts on sensing results as well. For example, in practical sensing processes, the overshooting phenomenon is a common situation in which external diffusion plays a vital role. Both external and internal diffusions result in a loss in actual gas concentration and have negative impacts on gas responses. The concept of the utility factor in internal diffusion can be further extended to external diffusion in future modeling. In addition, the linear assumption in diffusion models should also be altered to fit the power law. These approaches could be used for achieving a more vivid description of real sensing processes. ii) For the synthesis of sensing materials, surface modification is a promising way towards enhancing the adsorption/desorption of gas molecules. For example, surface alkalinity can be tuned by doping the framework of MOS using heteroatoms of metal elements, such as rare earth. The active sites of such materials are primarily the cations with non‐highest valences (e.g., Ce^3+^), which are common Lewis bases that can enhance the adsorption of acidic gas molecules. Similarly, the introduction of hydrophobic ingredients might eliminate humidity effects and enhance the adsorption of hydrophobic gas molecules. In addition, the synthesis of MOS materials with various morphologies and exposed crystal facets can contribute to gas adsorption as well. iii) The precise manipulation of pore structures (pore size, volume, crystallinity, wall thickness, and symmetry) of the sensing materials is critical to enhancing gas diffusion during the sensing process, and it can also promote higher sensing response and/or selectivity. Comparing the competitive rates between diffusion and reaction of each specific test gas, targeted regulation of pore structures can be expected. Several potential hypotheses are discussed as follows. For gas molecules with both high diffusivity and reactivity (e.g., H_2_), pore structures have fewer impacts on sensor response than on other gases. Approaches like reducing pore sizes or increasing layer thicknesses may help promote its selectivity, though the response value could be decreased to some extent. For gas molecules with low diffusivity and high reactivity (e.g., H_2_S), the diffusion impacts should be fully eliminated. MOS materials with larger pore sizes with lower layer thickness are a remedy for low sensor utility. On the other hand, the chemisorption and direct reaction between H_2_S and MOS sensing material can further enhance sensor response, while the recovery time may be shortened by surface modifications of the numerous oxygen vacancies in the pore wall. For gas molecules with high diffusivity and low reactivity (e.g., CH_4_), the sensor utility itself can be guaranteed. However, the reaction rate must be promoted. Generally, high‐performance noble metal catalysts like Pd, Pt, Au, Ag, Rh, and Ru are widely applied in the sensing and activation of light alkanes. In this case, the in situ doping of noble metals inside the mesopores through multicomponent coassembly (e.g., EISA) strategy is an ideal route. The gas molecules can interact with the huge interfaces of the porous sensing layer, promoting both utility and response. Moreover, manipulated pore morphologies of buckled‐cylindrical mesopores with large pore sizes and narrow window sizes are considered to be beneficial in enhancing diffusion into the mesopores and preventing releasing back, ensuring the enrichment of the gas molecules within the sensing layer for reaction promotion. For VOC molecules with extra‐low diffusivity and intermediate reactivity, the synthesis of 2D materials with extra‐high utility may help eliminate diffusion impacts. Moreover, the introduction of pulse‐driven gas sensors may be a significant addition to improving sensor response. Finally, the thickness of the sensing layer can be adjusted to fit a decent Hatta number according to the relative properties of the test gas molecules. In this case, the exploration of the sensing material needs to change from the conventional “material‐oriented” to a novel “gas‐oriented” one. Consequently, the intrinsic properties of MOS in terms of adsorption, desorption, and gas diffusion during gas sensing processes can be fulfilled by combining theoretically mathematical and physical considerations and the precise material design (e.g., modulating compositions and facets, engineering pore size and structure), and this marks a critical direction toward the future of MOS gas sensors.

## Conflict of Interest

The authors declare no conflict of interest.
